# Multiplexed spatial transcriptomics methods and the application of expansion microscopy

**DOI:** 10.3389/fcell.2024.1378875

**Published:** 2024-07-22

**Authors:** Andra Fortner, Octavian Bucur

**Affiliations:** ^1^ Medical School, Ruprecht-Karls-Universität Heidelberg, Heidelberg, Germany; ^2^ Victor Babes National Institute of Pathology, Bucharest, Romania; ^3^ Genomics Research and Development Institute, Bucharest, Romania

**Keywords:** spatial omics, transcriptomics, expansion microscopy, FISSEQ, ExSeq, MERFISH, Visium, Ex-ST

## Abstract

While spatial transcriptomics has undeniably revolutionized our ability to study cellular organization, it has driven the development of a great number of innovative transcriptomics methods, which can be classified into *in situ* sequencing (ISS) methods, *in situ* hybridization (ISH) techniques, and next-generation sequencing (NGS)-based sequencing with region capture. These technologies not only refine our understanding of cellular processes, but also open up new possibilities for breakthroughs in various research domains. One challenge of spatial transcriptomics experiments is the limitation of RNA detection due to optical crowding of RNA in the cells. Expansion microscopy (ExM), characterized by the controlled enlargement of biological specimens, offers a means to achieve super-resolution imaging, overcoming the diffraction limit inherent in conventional microscopy and enabling precise visualization of RNA in spatial transcriptomics methods. In this review, we elaborate on ISS, ISH and NGS-based spatial transcriptomic protocols and on how performance of these techniques can be extended by the combination of these protocols with ExM. Moving beyond the techniques and procedures, we highlight the broader implications of transcriptomics in biology and medicine. These include valuable insight into the spatial organization of gene expression in cells within tissues, aid in the identification and the distinction of cell types and subpopulations and understanding of molecular mechanisms and intercellular changes driving disease development.

## 1 Introduction

**FIGURE 1:**
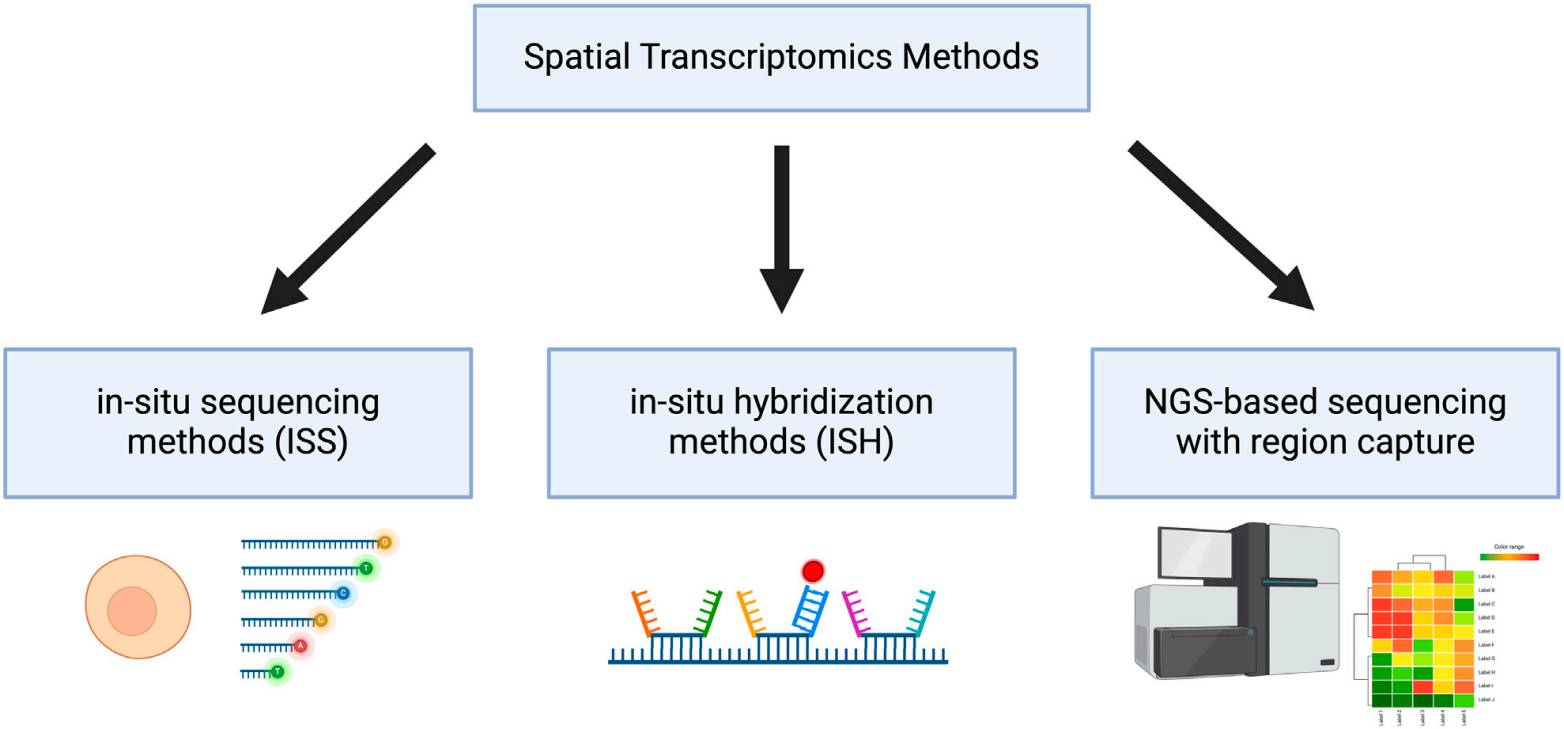
Classification of Spatial Transcriptomics Methods Spatial transcriptomic methods can be categorized into *in situ* sequencing methods (ISS), *in situ* hybridization methods (ISH) and next-generation sequencing (NGS)-based sequencing with region capture. Created with BioRender.com.

With the advent of omics, we have gained the ability to study cells in a more holistic approach, opening new insights into cellular heterogeneity, cell function, interactions and responses to stimuli (T. Y. Chen et al., 2023; Kolodziejczyk et al., 2015; Patel et al., 2014; Vandereyken et al., 2023). Referring to the Transcriptomics subdiscipline, which studies all the RNA present in a cell at a specific time, great advances have been made to reveal information about the expression levels of genes. Whereas in the past, only standard methods such as Real Time Quantitative PCR (RT-qPCR) and Northern blotting could be used, we can now apply novel transcriptomics techniques. With the help of transcriptomics methods, valuable information about all or most actively expressed genes and their tissue-specific and intercellular variations can be disclosed (Mistry et al., 2020). Furthermore, spatial transcriptomics additionally provides the locations of the certain RNAs detected in the cell. This can be done by using multiplex imaging approaches, i.e., high-throughput imaging of large numbers of RNAs. During the last years, new transcriptomics methods have appeared and have been applied to characterize cells, study the development and the biological basis of diseases. A broad classification of these methods can be made dividing them into *in situ* sequencing (ISS) methods, *in situ* hybridization (ISH) technologies and methods combining next-generation sequencing (NGS) with region capture (Figure 1) (Rao et al., 2021). Recently, many reviews have been published that give a great overview over the landscape of spatial transcriptomics methods, e.g., the review from Cheng et al., 2023.

Expansion Microscopy (ExM) is a super resolution and tissue clearing technique using physical tissue expansion to overcome the resolution limit of the standard light microscope (Tillberg et al., 2019). Whereas the resolution of a light microscope alone is maximum 250 nm, structures as small as 70 nm can be detected using expansion microscopy with the same microscope, after physical expansion (Zhao et al., 2017). The ExM technique has been developed in 2015 by Chen et al. and further optimized for multiple purposes, including for Pathology. Expansion Pathology (ExPath) has been introduced to facilitate the transition of the method into the clinical context (F. Chen et al., 2015; Bucur et al., 2020; Bucur and Zhao, 2018). ExM is based on four essential steps: (1) labeling biomolecules for later crosslinking to the gel (2) polymerization of a gel inside cells (3) the digestion of cellular proteins using Proteinase K to enable (4) expansion of the gel by the addition of water, which swells and thus magnifies space between the cellular compounds.

In this paper, we explore some innovative transcriptomics methods that have been combined with ExM protocols and investigate how these methods have been improved by the use of ExM.

## 2 Methods in multiplexed spatial transcriptomics

### 2.1 *In situ* sequencing methods

In *in situ* sequencing (ISS) methods sequencing is directly carried out on the single-cell or tissue. In order to perform these methods, complementary DNA (cDNA) first needs to be synthesized from the RNA *in situ*, which can then be used for the sequencing (Vandereyken et al., 2023). ISS can either target specific RNAs or it can sequence the RNA present in cells in an untargeted approach (Rao et al., 2021; Vandereyken et al., 2023). An advantage of untargeted approaches is that they enable identification of the variability of RNAs depending on their location in the cell and the detection of splicing differences as well as accumulation of excised introns (Morgan et al., 2019; Alon et al., 2021). Since the first use of ISS by Ke et al., in 2013 who examined the expression of target genes in breast cancer, this technology has been adapted and further developed (Ke et al., 2013). Lee et al. (2014) introduced fluorescent *in situ* RNA sequencing (FISSEQ) being the first untargeted approach to transcriptomics (Lee et al., 2014).

#### 2.1.1 Fluorescent *in situ* RNA sequencing (FISSEQ)

Fluorescent *in situ* RNA sequencing (FISSEQ) sequences RNAs directly in a cell creating quantitative information about the amount of specific RNAs throughout the cell and qualitative data, i.e., the sequence of the RNAs which can be then used for further analysis.

After fixation and permeabilization of the cells, the method involves reverse transcription of the RNA to cDNA within the cell, accompanied by the addition of aminoallyl dUTP molecules which incorporate into the cDNA (Lee et al., 2015). Subsequently, critical adaptors needed for the later sequencing step are ligated to the ends of the cDNAs. A chemical reaction then cross-links the cDNA template to the protein matrix of the cell to prevent locational change due to diffusion. RNA degradation follows to remove competitive inhibitors of CircLigase, an enzyme catalyzing the ligation of single stranded DNA into circles.

**FIGURE 2:**
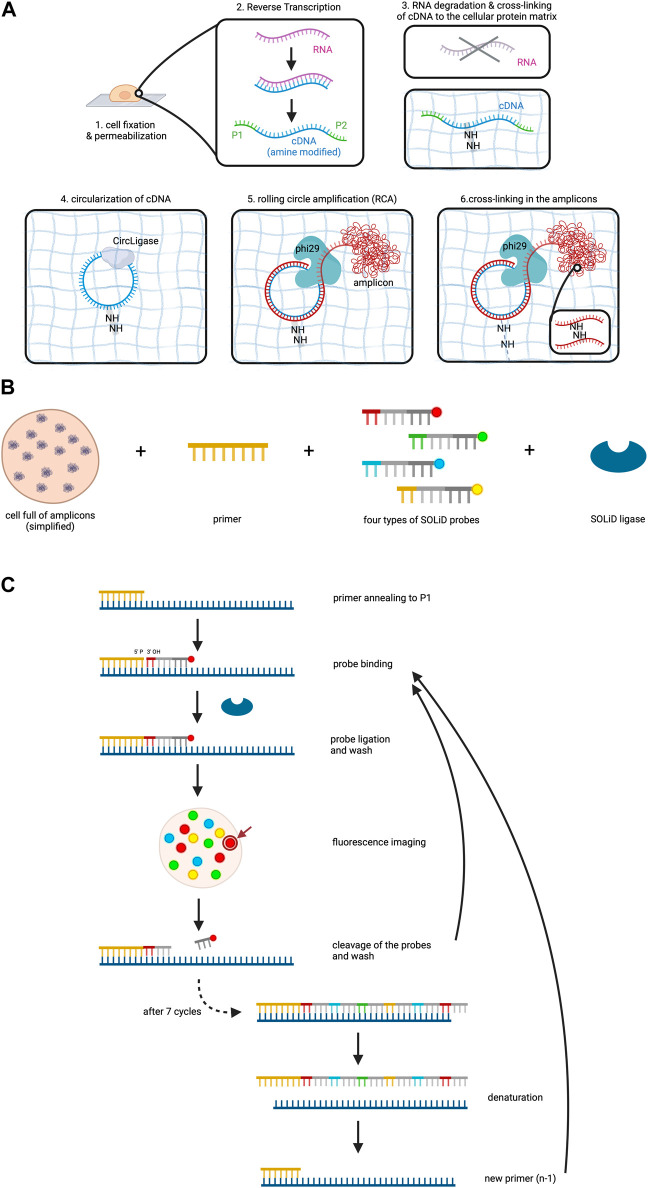
FISSEQ. The figure depicts the FISSEQ method for spatial transcriptomics. **(A)** library preparation for *in situ* sequencing. After fixing the cell on a glass slide and making it permeable with Triton X100, reverse transcription of the RNA is performed. A cDNA strand is synthesized from the RNA, which is amine-modified by the addition of aminoallyl dUTP during synthesis. The P1 and P2 adaptors are attached to the respective ends of the cDNA, which is needed for later sequencing steps. The RNA is then degraded and the primary amine groups of the aminoallyl dUTPs are used to anchor the cDNA in place on the cellular protein matrix. The cDNA is circulated by CircLigase in order to perform rolling circle amplification on the cDNA. Thus, a long strand of DNA is created which is called an amplicon and is cross-linked in the next step. **(B)** key reagents needed for SOLiD sequencing. All RNA in a cell has been prepared and is now available for sequencing. The copies of the initial RNA are bordered by P1 and P2 adaptors as explained above. Furthermore, a primer complementary to the P1 adaptor, four types of probes and the SOLiD ligase are needed. **(C)** SOLiD sequencing mechanism. After annealing the primer to the DNA template, the fitting probe binds to the template and is ligated to the primer by SOLiD ligase. The color of the fluorescent dye can be detected during imaging at the specific location in the cell. The arrow gives an example of where the fluorescent dye used in this figure may be visualized in the cell under the microscope. This is followed by cleavage to remove the fluorescent probes. The next cycle starts again with the addition of probes. After seven cycles the newly synthesized strand is removed and a new primer that is one base shorter than the first one is added. The whole procedure is repeated until in total five rounds have been completed, i.e. 5 different primers have been used. Created with BioRender.com.

Circularized cDNA serves as a template during rolling circle amplification (RCA), a technique for nucleic acid amplification which in contrast to the widely used polymerase chain reaction (PCR) operates isothermally and uses a circular DNA as a template (Garafutdinov et al., 2021). RCA involves three steps: (1) Annealing of a starting primer to the single-stranded circular DNA template, (2) synthesis of the complementary strand along the circle by a special polymerase with strand displacement activity and (3) displacement of the copy from the circular DNA template as soon as the 5’ end of the primer is reached, while at the same time continuing the synthesis along the circle (Garafutdinov et al., 2021). This procedure thus creates one long single-stranded DNA which can contain between 10 to a few thousand copies of the template (Lee et al., 2015; Garafutdinov et al., 2021). In this experiment aminoallyl dUPTs are also used during RCA and cross-linking is again performed in order to form the long amplified DNA copy into a tuft called the RCA amplicon (Figure 2A). RCA amplicons typically measure around 200–400 nm in diameter (Lee et al., 2014). While this small size allows for dense localization within the cellular context, the high abundance of these amplicons can lead to optical crowding. Optical crowding can potentially impact the accuracy of FISSEQ by making it difficult to resolve closely spaced transcripts. Proper optimization and careful interpretation of the data are essential to mitigate these issues and ensure precise RNA localization and quantification.

Sequencing utilizes sequencing by oligonucleotide ligation and detection (SOLiD) which is carried out at room temperature under a confocal microscope (Lee et al., 2015). SOLiD, invented at the George Church laboratory at Harvard Medical School in 2005 and subsequently improved and commercialized upon 2 years later by Applied Biosystems necessitates cDNA fragments flanked by P1 and P2 adaptors on opposite ends, a step which has already been addressed during reverse transcription of the RNA (Shendure et al., 2005; Voelkerding et al., 2009). Primers are introduced, annealing to the P1 adaptor and providing a 5′ phosphate group for ligation (Voelkerding et al., 2009; Lee et al., 2015). Next, eight nucleotide long probes are added which are attached to a fluorescent dye. These probes are made up of four distinct parts which from 3′ to 5′ are: (1) a combination of two bases, e.g., AT, CT, GG. As there are four bases, a permutation of 16 bases is possible. (2) three degenerate bases able to bind to any of the four bases A, T, C and G. (3) three inosine bases and (4) a fluorescent dye (Lee et al., 2015; Voelkerding et al., 2009; ATDBio - Next-Generation Sequencing, n.d.).

Four types of probes, each with a unique fluorescent dye color corresponding to specific base combinations, are employed (Voelkerding et al., 2009). SOLiD ligase is then added to initiate sequencing (Figure 2B). The process involves primer binding to P1, complementary probe annealing to the DNA fragment adjacent to the primer, and SOLiD ligase joins the 5′ phosphate group of the primer with the 3′ hydroxyl group of the probe. After a washing step, the fluorescent signal of the probe is measured, which is followed by cleavage of the fluorescent dye including the adjacent three inosine bases of the probe, resulting in a five-base-long remaining probe (Voelkerding et al., 2009; Lee et al., 2015). Seven cycles of probe annealing, ligation, washing, fluorescence measurement and washing are repeated.

To this point, the data collected only comprises the fluorescent measurement for each fifth base on the DNA fragment. In order to identify the bases that lie in between, denaturation is performed to remove the newly synthesized strand, followed by the addition of a new primer off-set by one base (n-1) (*ATDBio - Next-Generation Sequencing*, n.d.; Lee et al., 2015; Voelkerding et al., 2009). In total, five cycles are performed (n, n-1, n-2, n-3, n-4), ensuring data collection for each base on the DNA fragment and even more so, it means that each base of the DNA fragment has been read out twice (Figure 2C).

The data generated by SOLiD sequencing is next interpreted by analyzing the colors emitted in the five rounds. Therefore, the 3D cell images are processed by 3D deconvolution techniques and MATLAB to precisely overlay colors and convert those into TIFF images. Thus, pixel colors are analyzed to deduce the base sequence using Python. This base sequence is then compared to the reference transcriptome (Lee et al., 2015). In the Python sequence analysis, it is taken into consideration that each color corresponds to four distinct base combinations. By integrating information about the base’s position on the sequenced DNA inferred from the round and cycle the color reading had been obtained in, and an algorithmical decoding of the color’s bases, the sequence of the DNA template and thus of the initial RNA can be determined (Valouev et al., 2008; Voelkerding et al., 2009). Ultimately, the parameters obtained by data analysis encompass various aspects such as “the number of individual pixels per object, gene ID, consensus sequence, x and y centroid positions, number of mismatches, base call quality and alignment quality” (Lee et al., 2015).

#### 2.1.2 Expansion sequencing (ExSEQ)

Alon et al. have combined *in situ* sequencing with the method of expansion microscopy (ExM) to enable high-resolution imaging of RNA in the subcellular compartments (Alon et al., 2021). Expansion sequencing (ExSEQ) is based on FISSEQ but offers better accessibility to the RNA inside the cell as the amplicons become less dense, and more precise spatial mapping (Wassie et al., 2019; Alon et al., 2021). The experiment starts with tissue fixation and permeabilization, followed by RNA anchoring and the alkylation of RNA bases with LabelX to anchor RNAs to the cellular matrix (Alon et al., 2021).

**FIGURE 3:**
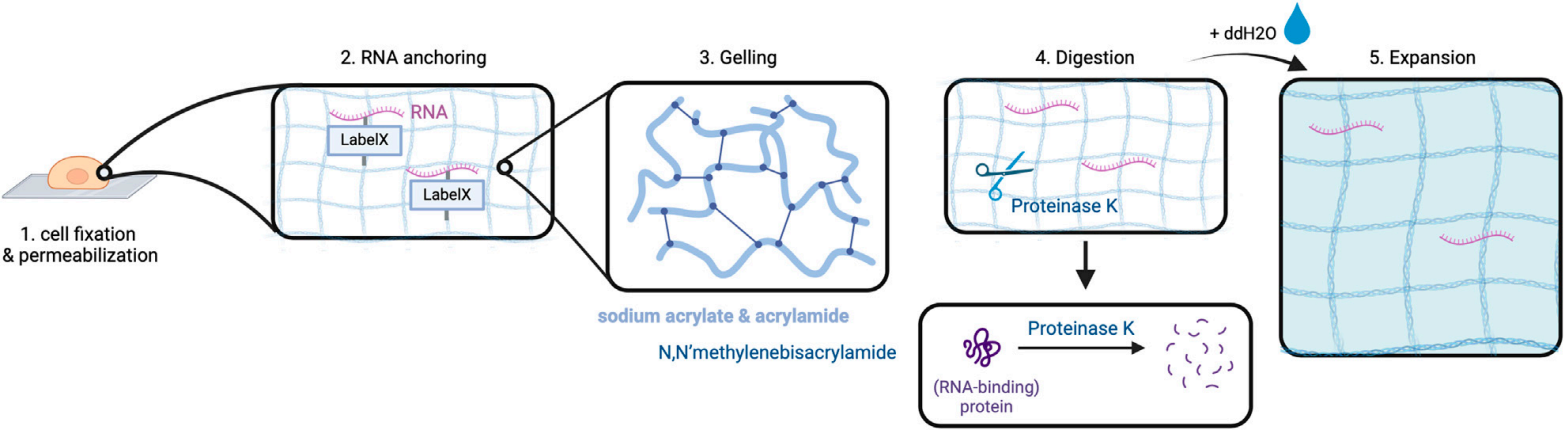
Expansion in ExSEQ. After fixing and permeabilizing the cell (1) RNA anchoring: cellular biomolecules including RNA are labeled for later anchoring to the gel, which is achieved by covalently binding the LabelX to the RNA and the protein matrix (2). The cell is placed in a gel chamber, in order for the gelling solution to polymerize (3). Proteinase K cleaves the protein matrix of the cell into pieces (4) that can then spread isotropically after the addition of water, depicted as a light blue background in (6). Created with BioRender.com.

This is a critical step to prepare the biomaterial ensuring even distribution of the cross-linked RNAs along with cell matrix molecules during the later expansion step. Subsequently, cells are put into a gelling solution and then transferred into a gel chamber where radical polymerization takes place (Alon et al., 2021). This is followed by digestion carried out in overnight incubation with Proteinase K which is responsible for protein cleavage and release of nucleic acids (Alon et al., 2021). Lastly, expansion is performed by incubation of the cells in double-distilled water (ddH2O) (Alon et al., 2021). The purpose of digestion is to prepare the cells for expansion as it enables the cleaved proteins, especially structural proteins, to spread in an isotropic way during expansion. At this point, an expansion of approximately 3.3x of the cells can be obtained (Figure 3) (Alon et al., 2021).

The next step is re-embedding, i.e., the expanded gel is placed into a polyacrylamide gel that does not expand (Alon et al., 2021). This step is necessary to ensure that the expanded gel does not change its conformation further, because it would falsify the results during RNA sequencing (Alon et al., 2021). Because of an inhibition of enzymes needed for later sequencing by carboxylic acid groups of sodium acrylate in the expansion gel, passivation is required (Alon et al., 2021). During passivation, ethanolamine forms a covalent bond to the carboxylic groups which results in a chargeless amide (Alon et al., 2021).

From here, the experiment continues with the FISSEQ procedure. More precisely, the RNA is reverse transcribed and then removed, cDNA is circularized and RCA is performed (Alon et al., 2021). Prior to these steps, the cell’s genome is digested by DNAse I to avoid contamination of the cDNA (Alon et al., 2021). As the ExM protocol is conducted before the RCA step, the size and density of RCA amplicons remain unchanged when compared to the standard FISSEQ procedure. However, the increased distance between neighboring replicons enhances RNA resolution. Lastly, SOLiD sequencing using confocal microscopy can be carried out to obtain the data, which can then be interpreted (Alon et al., 2021).

#### 2.1.3 Other *in situ* sequencing approaches

Examples of other ISS methods are ISS using padlock probes (Ke et al., 2013) and STARmap (spatially resolved transcript amplicon readout mapping) (X. Wang et al., 2018), which are both targeted approaches for ISS. FISSEQ in contrast to these other two methods is untargeted and does not use padlock probes. Padlock probes are DNA oligonucleotides whose ends are complementary to two adjacent parts of a targeted RNA or DNA sequence. The padlock probes’ 5′ and 3′ end sequences are designed in a way, so that the padlock probe oligonucleotide forms into a circle when those end sequences bind to the targeted RNA or DNA respectively. The padlock probe is then used to perform RCA on the target sequence to create the amplicons needed for sequencing. Additional to the untargeted ExSEQ method described above, Alon et al. also presented a targeted approach of ExSEQ which is based on barcoded padlock probes that hybridize to the target RNAs. RNA detection then occurs by sequencing the barcodes (Alon et al., 2021).

Although STARmap also embeds the tissue in a hydrogel, it does not do that with the purpose of performing expansion on the tissue, but rather to optimize imaging conditions, as the induction of the gel into the tissue ameliorates transparency and decreases background noise. Hence, tissue expansion by ExM technology has not yet been used on these ISS approaches which is why we do not go into further detail here.

### 2.2 *In situ* hybridization methods

Fluorescence *in situ* hybridization (FISH) is a widely used method with many applications in molecular biology and diagnostics. By delivering fluorescent labeled probes into a cell which hybridize to a complementary target sequence, the detection of specific DNA or RNA sequences in cells is made possible (Bayani and Squire, 2004). This procedure has been adapted to enable transcriptome-wide analyses. Firstly, single-molecule FISH (smFISH) methods have emerged visualizing and quantifying RNA in fixed cells by hybridization of cellular RNA to a number of different FISH probes containing colored fluorescent dyes (J. Chen et al., 2018; Lubeck et al., 2014; Lubeck and Cai, 2012; Raj et al., 2008). However, only a limited number of RNAs (ca. 30) could be studied by these methods at a time because of the restricted number of fluorescence channels for the fluorophores (Zhuang, 2021). Recently, advances have been made to increase throughput of ISH methods and lead to the development of the methods MERFISH and RNA seqFISH. The main difference between these methods lies in the barcoding to infer RNA identity: While MERFISH uses a binary barcode to identify RNA types, RNA seqFISH encodes RNAs through color-schemed barcodes.

#### 2.2.1 Multiplexed error-robust fluorescence *in situ* hybridization (MERFISH)

MERFISH is an innovative method employing combinatorial labeling with high throughput imaging and error-robust barcoding. When it was first introduced by Chen et al., in 2015 it enabled the simultaneous analysis of about 1,000 genes (K. H. Chen et al., 2015). This capacity has been enhanced by integrating MERFISH with ExM to address more than 10.000 genes in one single MERFISH experiment (see 2.2.2). However, in contrast to ISS methods, ISH technology can only be performed in a targeted approach because knowledge about the RNA types in a cell to be identified in the ISH method is a prerequisite to encode the FISH probes. MERFISH enables location, identification and quantification of RNA types of the transcriptome. To avoid misunderstandings, it should be clarified that the terms “RNA type” and “gene” are used synonymously in this article. However, we more frequently use the term “RNA type” as it emphasizes the RNA nature of the molecules under consideration.

The key aspect of MERFISH lies in the assignment of each targeted cellular RNA type to a specific binary barcode composed of the digits (bits) 0 and 1. These digits indicate if the specific RNA type emits a fluorescent color during the respective imaging round corresponding to the bit one in the code, or if no color will be measured by the fluorescence microscope which is encoded as the bit 0. For example, the barcode 100 means that in the one particular spot on the images, i.e., the same location within the cell, a fluorescence signal will be measured in the first imaging round, but in round two and three the spot will remain dark. In order to achieve this alternating light emission for a specific RNA type in the different imaging cycles, the design of so-called encoding probes is essential. These encoding probes hybridize with high specificity to the RNA type which they have been designed for. Additionally to the RNA type specific sequence, the encoding probes contain certain sequences which serve as binding spots for oligonucleotides linked to a fluorescent dye. The color emission observed under the microscope is then achieved by the addition and removal of the fluorescent dye-linked oligonucleotides in each round which can either bind to the encoding probe of a specific RNA type or not.

However, one aspect that has to be considered is the proneness to error: a misreading of 1 bit alone produces a falsified binary barcode that matches to a different RNA type (K. H. Chen et al., 2015). Thus, the RNA type that is actually located in a particular spot would be mistaken for another RNA type just because of an error in one imaging cycle. This also clarifies that the risk of error increases with the amount of MERFISH cycles carried out (K. H. Chen et al., 2015). To address this, smartly designed, so-called error-robust barcodes can be generated by introducing a Hamming distance for all the barcodes (K. H. Chen et al., 2015). Hamming distance is a concept in informatics which describes the number of positions within a string that differ to all the other strings in the same data set. For instance, at a Hamming distance of 4, 4 bits can be changed by errors without resulting in a barcode that encodes another RNA type. Instead, the error in the barcode can be detected and changed by the programme to enable correct RNA type identification (K. H. Chen et al., 2015).

Thus, to start the experiment, the data on RNA types/genes to be targeted in the MERFISH experiment including their sequences are retrieved from databases, in order to assign each gene to their respective error-robust barcode and to generate specific encoding probes (K. H. Chen et al., 2015; Moffitt et al., 2016). Each encoding probe consists of the following components: (1) a target-binding region in the center for hybridization to the specific RNA type, (2) different readout sequences bordering the target-binding region for fluorescent dye binding, (3) primers framing the readout sequences to both sides to enable encoding probe amplification. The number of encoding probes depends on the number of RNA types to be sequenced. Generally, the more encoding probes per RNA type are used, the stronger is the potential fluorescence signal under the microscope.

**FIGURE 4:**
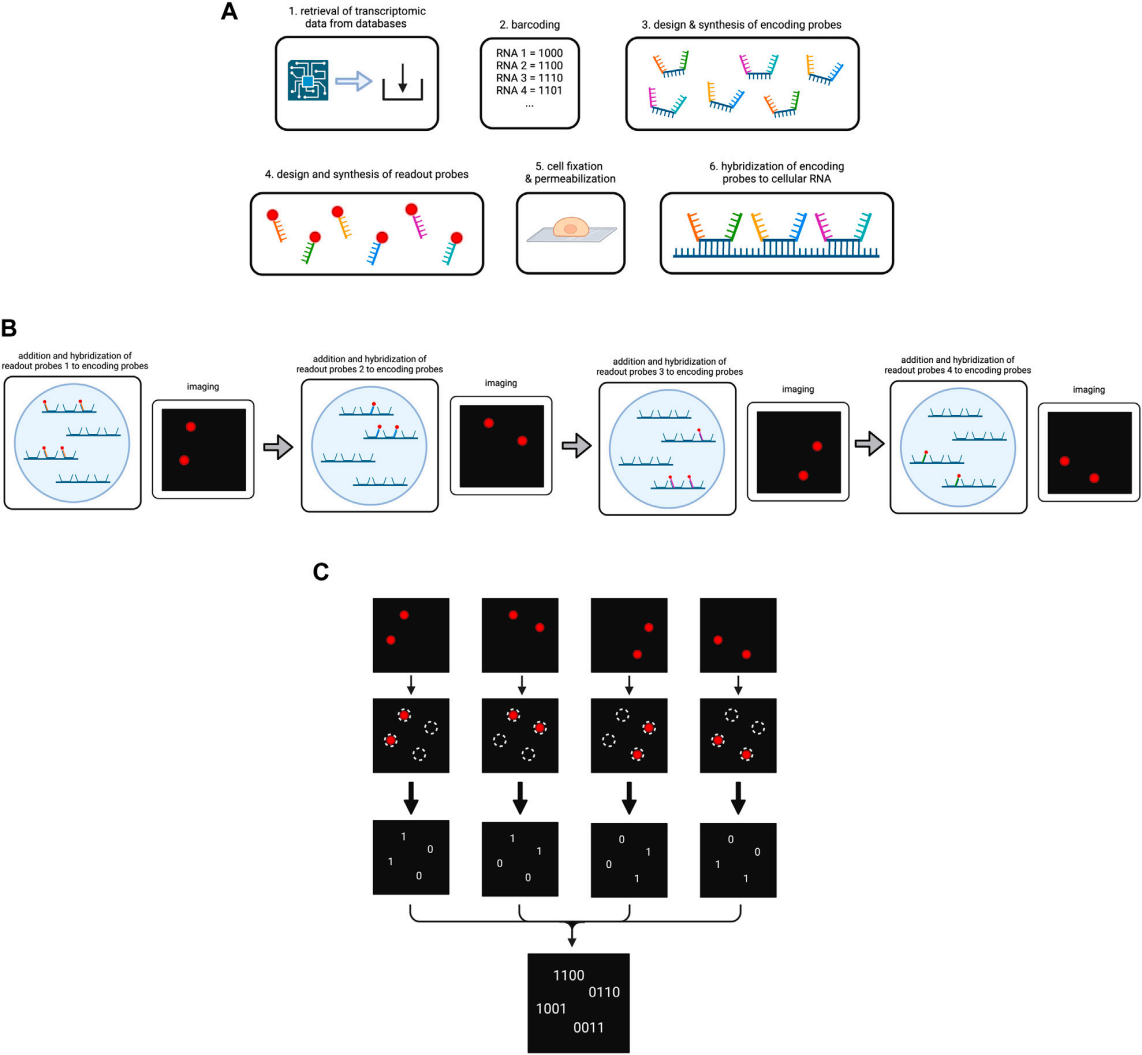
Overview on the MERFISH method. **(A)** Preparation of the cellular RNA library. Targeted RNA types are determined and encoded by a barcode each. The encoding probes and readout probes are designed and synthesized. After cell fixation and permeabilization encoding probes are added to the cells, so they hybridize to the cellular RNA. **(B)** Simplified scheme of the operating principle of MERFISH cycles. For the purpose of simplification, our cell contains four RNAs that have been hybridized to encoding probes. We use four different readout probes in four MERFISH cycles. In each cycle a different type of readout probe is added which hybridizes to the respective readout sequences on the encoding probes. After hybridization, imaging occurs and shows the RNAs that have bound to the respective readout probe. The fluorescence is then removed for the next cycle to start. **(C)** Decoding of the MERFISH images. The single images from each MERFISH cycle are analyzed to determine the spots in which targeted RNAs are located. A present or lacking fluorescent signal in the respective spot is transformed into the bits 1 or 0. Combining the bits from each MERFISH cycle, the binary code is obtained that indicates which RNA type is located in the respective spot. Created with BioRender.com.

Referring to the readout sequences on the encoding probes, homology to cellular RNA has to be avoided in order to prevent errors during hybridization (K. H. Chen et al., 2015). Each specific readout sequence corresponds to one cycle of hybridization and imaging, and is complementary to a readout probe, which is composed of an oligonucleotide attached to the fluorescent dye. Following synthesis of the encoding probes and readout probes, cells are immobilized on a coverglass and permeabilized before exposure to encoding wash buffer (K. H. Chen et al., 2015; Lee et al., 2014). Encoding probes in a hybridization buffer are then pipetted on a microscopic slide which is next covered with the cell-coated coverglass (K. H. Chen et al., 2015; Moffitt et al., 2016). The cells are then incubated to enable hybridization (Figure 4A). Subsequent steps involve washing, incubation with fluorescent beads for later image alignment and fixation of the sample with paraformaldehyde (K. H. Chen et al., 2015).

At this point, MERFISH cycles can start, which are carried out in a flow chamber under the fluorescence microscope. In each round, the sample is hybridized with readout probes, imaged for fluorescent light emission and decolorized by removal of the fluorescent dye (Figure 4B). In more detail, one type of readout probes per MERFISH cycle dissolved in a hybridization buffer is infused into the chamber. During incubation the readout probes hybridize to the corresponding readout sequences on some of the encoding probes. The flow chamber then washes the sample and imaging occurs for two color channels: one for the readout probes and one for the beads (X. Chen et al., 2019). In order to remove the fluorescent light emission, either photobleaching or cleavage of the fluorescent dye, e.g., by TCEP (tris(2-carboxyethyl)phosphine) can be performed, and the samples are imaged again to check if the decolorization was successful (K. H. Chen et al., 2015; Moffitt et al., 2016). The number of cycles has been determined during the barcoding of the RNA types as it corresponds to the numbers of bits in the codes.

Upon completion of all cycles, image alignment is performed with the help of the fluorescence signals by the small ​​fluorescent beads in each image and a coordinate system is established for data analysis (K. H. Chen et al., 2015). A multi-Gaussian-fitting algorithm is used to identify RNA location from fluorescence signals (K. H. Chen et al., 2015). For instance, it can detect overlapping readings in a pixel and correctly assign them to two different individual RNA. Each image is analyzed for whether a fluorescence is measured at a specific location (1) or not (0) and the bits are noted to obtain the final binary code for the respective RNAs (Figure 4C) (K. H. Chen et al., 2015). From the obtained binary codes, the RNA type in each of the locations can be concluded. The MERFISH procedure is depicted in Figure 4.

The MERFISH method can be further enhanced to study a broader range of genes simultaneously firstly by increasing barcode length and thus number of readout probes and hybridization rounds, and secondly by using more fluorescent colors channels per hybridization cycle (Moffitt et al., 2016). Hence, the total number of possible RNA types that can be studied in a MERFISH experiment can be theoretically calculated by multiplying the number of hybridization rounds (N) which equals the numbers of digits per barcode, with two because each digit has two possible outcomes (1 or 0) which in turn is multiplied with the amount of colors used (C): 2NC = number of RNA types distinguishable in the MERFISH experiment (Zhuang, 2021).

#### 2.2.2 Application of expansion in MERFISH

Recently, performance of the MERFISH method has been improved by combination with ExM (Wang et al., 2018; Xia et al., 2019). Application of the ExM technology decreases RNA density which significantly improves resolution, allowing for more genes to be studied in a MERFISH experiment. Hence, in 2019, Xia et al. demonstrated the detection and identification of 10.050 genes using a total of 69 readout probes and three fluorescent dyes across 23 hybridization cycles performed (Xia et al., 2019).

The procedure is very similar to the MERFISH method by Chen et al. and starts with the creation of error-robust barcodes, design and synthesis of encoding probes and readout probes similarly as described above (Xia et al., 2019). Besides, labeling of cellular structures, e.g., the nucleus and endoplasmic reticulum (ER) can be carried out to better characterize the location of RNA spots in the cell (Xia et al., 2019). To do so, the stain DAPI can be used for nuclear visualization, while KDEL can be used to label the ER. Cells are immunostained after cell fixation and permeabilization to a coverglass (Xia et al., 2019). Immunostaining includes a primary antibody binding to subcellular structures and a secondary antibody which is conjugated with oligonucleotides that can later hybridize to fluorescent complementary probes that can be visualized in an imaging round before the start of the MERFISH cycles (Xia et al., 2019). The antibody labeling is fixed with PFA to avoid their detachment in the further procedure. The experiment continues with hybridizing the encoding probes to the cellular RNA. However, to prepare the cellular RNA for expansion, acrydite-modified poly(dT) locked nucleic acid (LNA) probes are added into the hybridization solution, where they hybridize to the poly(A) tails of mRNAs which enables anchoring to the expansion gel (Wang et al., 2018; Xia et al., 2019). This is followed by incubation with small beads that are to be used for location reference to align multiplexed images in a later step as previously described (Figure 3).

For ExM, the cells are firstly incubated with a gel solution and polymerization is started. Digestion is carried out by placing the sample into a solution of sodium dodecyl sulfate (SDS), Triton X-100, Proteinase K and SSC overnight (Xia et al., 2019). Then, isotropic expansion is performed in saline sodium citrate (SSC) buffer, followed by re-embedding of the gel in a polyacrylamide gel to prevent conformational changes of the expanded gel (Xia et al., 2019).

The MERFISH cycles take place in a flow chamber and consist of the three procedures mentioned above: readout probe hybridization, imaging and fluorescence removal. In more detail the steps as described by Xia et al. are: (1) addition and hybridization of three encoding probes per cycle to the cellular RNA, (2) washing, (3) imaging, (4) removal of the fluorescent dye and (5) washing (Xia et al., 2019). Imaging is carried out at wavelengths corresponding to the three fluorescent dyes used, revealing three digits of the individual barcodes per cycle (Xia et al., 2019).

Once the data has been obtained image analysis is performed by aligning the images with the help of the signals from the beads, identifying fluorescence spots that indicate RNA location and transforming the light signals into bits to obtain the barcodes by a voxel-based decoding algorithm (Xia et al., 2019). Hence, the RNAs detected can be identified and counted to compare the amount of each RNA type. Additionally, as spatial relations are preserved the accumulation of RNA types in certain subcellular compartments can be examined.

### 2.3 NGS sequencing with region capture

In contrast to ISS and ISH technologies, RNA in NGS-based transcriptomic methods is physically extracted from the cells. After reverse transcription of the RNA into cDNA next-generation sequencing is performed to discover the identity of the RNA while the location can be traced back by the individually assigned barcode of the respective RNA. Hence, NGS-based sequences are not carried out *in situ* but require region capture and reconstruction of the spatial data after sequencing.

#### 2.3.1 VISIUM

**FIGURE 5:**
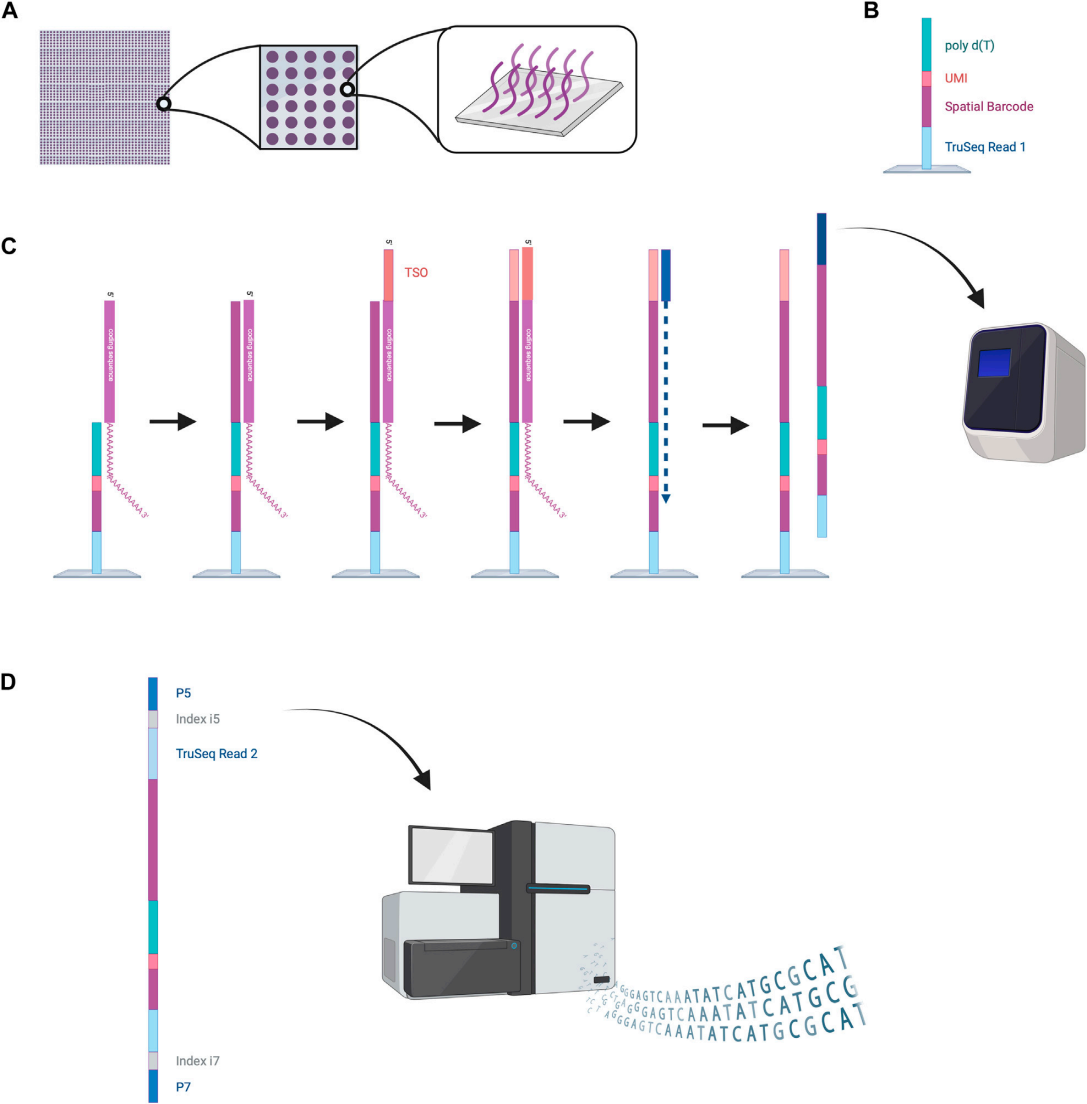
NGS-based sequencing with region capture by the VISIUM platform. **(A)** A capture area consists of 5,000 small spots in which capture probes are fixed to the bottom of the wells. **(B)** Structure of the capture probes. **(C)** mRNAs are captured inside the spots by the capture probes. Reverse transcription is performed, and second strand synthesis provides cDNA that is then amplified by PCR. **(D)** Amplified oligonucleotides are prepared for Illumina sequencing by the attachment of various oligonucleotides (TruSeq Read two sequence, indices, P5 and P7). These can be processed by the Illumina sequencer to read out the spatial barcode and UMI for spatial mapping and the RNA coding sequences for RNA identification. This data enables reconstruction of the cellular RNA map. Created with BioRender.com.

The VISIUM platform stems from the works of Ståhl et al., in 2016 (Ståhl et al., 2016). They introduced the first NGS-based transcriptomic method for transcriptome-wide analysis which they named “Spatial Transcriptomics” and was later commercialized by 10x Genomics to create the VISIUM platform. VISIUM transfers mRNAs from tissue sections onto a microarray with the help of capture probes and assigns each well to a specific barcode. A capture area (microarray of 6.5 × 6.5 mm) in VISIUM contains 5,000 spots. Each spot contains multiple fixed capture probes on the bottom that consist of Illumina TruSeq Read one at the 5’ (a sequencing primer for Illumina), a spatial barcode for tracing back the well-location of an mRNA, a unique molecular identifier (UMI) for tracing back the exact location of the mRNA inside the well and a poly(dT) primer for binding and thus capturing poly-adenylated mRNA (Hudson and Sudmeier, 2022) (Figure 5).

To start the experiment, a tissue slice is fixed on the microarray, stained with hematoxylin and eosin (HE) and an image is taken under a microscope (Hudson and Sudmeier, 2022). Cells are permeabilized to enable mRNA diffusion into the wells where mRNA is captured by the fixed capture probes. Inside these spots, reverse transcription is performed to obtain a cDNA oligonucleotide containing the illumina TruSeq 1 read, the spatial barcode, the UMI and the RNA-specific sequence (Hudson and Sudmeier, 2022). More precisely, the capture probe is firstly elongated with the RNA-specific complementary sequence and the 5′ end of the mRNA is extended with a template switch oligo (TSO) primer to prevent loss of information at the 5′ end. The TSO is then hybridized to a primer for complementary strand synthesis. After denaturation, cDNAs from each spot are transferred into separate tubes, amplified by PCR and prepared for sequencing (Hudson and Sudmeier, 2022). Preparation steps include the removal of the TSO, attachment of the TruSeq Read 2, and flanking with index regions. Lastly the cDNAs are flanked with P5 and P7 sequences, which will hybridize to the flow cell’s oligonucleotides during Illumina sequencing. The Truseq Read one and two are sequencing primer binding sites needed to perform sequencing-by-synthesis. The indexes work as identifiers to allow sequencing of multiple cDNA libraries. The last step is sequencing. Sequencing cycles reading Truseq Read one sequence the spatial barcode and UMI, whereas sequencing cycles reading Truseq Read two produce the data of the cDNA sequence (Hudson and Sudmeier, 2022). Utilizing the spatial barcode sequences, RNA locations in the tissue can be reconstructed providing valuable information about local gene expression patterns.

#### 2.3.2 Expansion spatial transcriptomics (Ex-ST)

A big disadvantage of VISIUM is the inability of obtaining single-cell transcriptomic resolution as the spots on the capture area each have a diameter of 55 μm, thus fitting more than 1 cell per spot and eventually mixing RNA data from different cell types (Fan et al., 2023). In 2023, Fan et al. combined the VISIUM platform with expansion to address this challenge and named this method “Expansion spatial transcriptomics” (Ex-ST). As expansion technology enables physical magnification of the tissue, cell size increases and mRNA availability is enhanced for region capture. It has been shown that when VISIUM is combined with ExM the cell number per spot is significantly decreased and resolution of VISIUM can be lowered from only 55 μm to 20 µm (Fan et al., 2023). Furthermore, the combination of these methods allows for more transcripts to be detected per normalized tissue area and conclusions about subcellular location of transcripts in certain types of cells, e.g., neurons, can be drawn (Fan et al., 2023). This brings valuable data to better characterize cell types and explore functional heterogeneity in tissues.

The experiment starts with fixation of cryosectioned tissue on a glass slide, which in contrast to the original VISIUM is followed by expansion. Before beginning the expansion procedure RNAs need to be anchored which is achieved by adding LNA to the hybridization buffer (Fan et al., 2023). The cells are gelled in the next step. Therefore the tissue is first incubated at low temperature in a monomer solution (PBS, NaCl, sodium acrylate, acrylamide, N,N′-methylenebisacrylamide) which is followed by radical polymerization upon the addition of APS along with TEMPO, tetramethylethylenediamine and 4-hydroxy-2,2,6,6,-tetramethylpiperidin-1-oxyl, a hindered amine light stabilizer used to stabilize the resulting polymer (Fan et al., 2023). Digestion is then performed overnight by Proteinase K diluted in digestion buffer, followed by gel expansion in SSC (Fan et al., 2023). At this point, the gel can be transferred onto the capture area of the VISIUM platform. Optionally, nuclei can be stained with DAPI before the transfer and imaged on the VISIUM microarray. Subsequent steps align with the standard VISIUM protocol, encompassing RNA release and capture by the capture probes, reverse transcription, second strand synthesis, cDNA amplification by PCR, cDNA processing for Illumina sequencing, and the placement of the VISIUM arrays into the Illumina sequencer for NGS to be performed (Fan et al., 2023). Modifications to the standard protocol were proposed by Fan et al. include adjustments to, e.g., reverse transcription temperature and the number of PCR cycles. The data obtained is processed by space ranger software from 10x Genomics where RNA transcripts are compared to the reference genome. The data can then be analyzed in Python and R to recreate spatial gene expression maps (Fan et al., 2023).

#### 2.3.3 Other NGS-based methods with region capture

Since the introduction of NGS-based approaches with region capture for spatial transcriptomics by Ståhl et al. many more similar methods have been developed to increase RNA detection yield. However, none of these methods except VISIUM have been previously combined with ExM protocols. Thus, the application of ExM on NGS-based methods with region capture offer a highly interesting research field with the potential of commercialization, given by its compatibility with next-generation sequencing technologies, which have become integral components of laboratory instrumentation in genetic research settings. Some examples for other NGS-based technologies with region capture include Slide-seq, Slide-seqV2, Stereo-seq and Seq-Scope.

**FIGURE 6:**
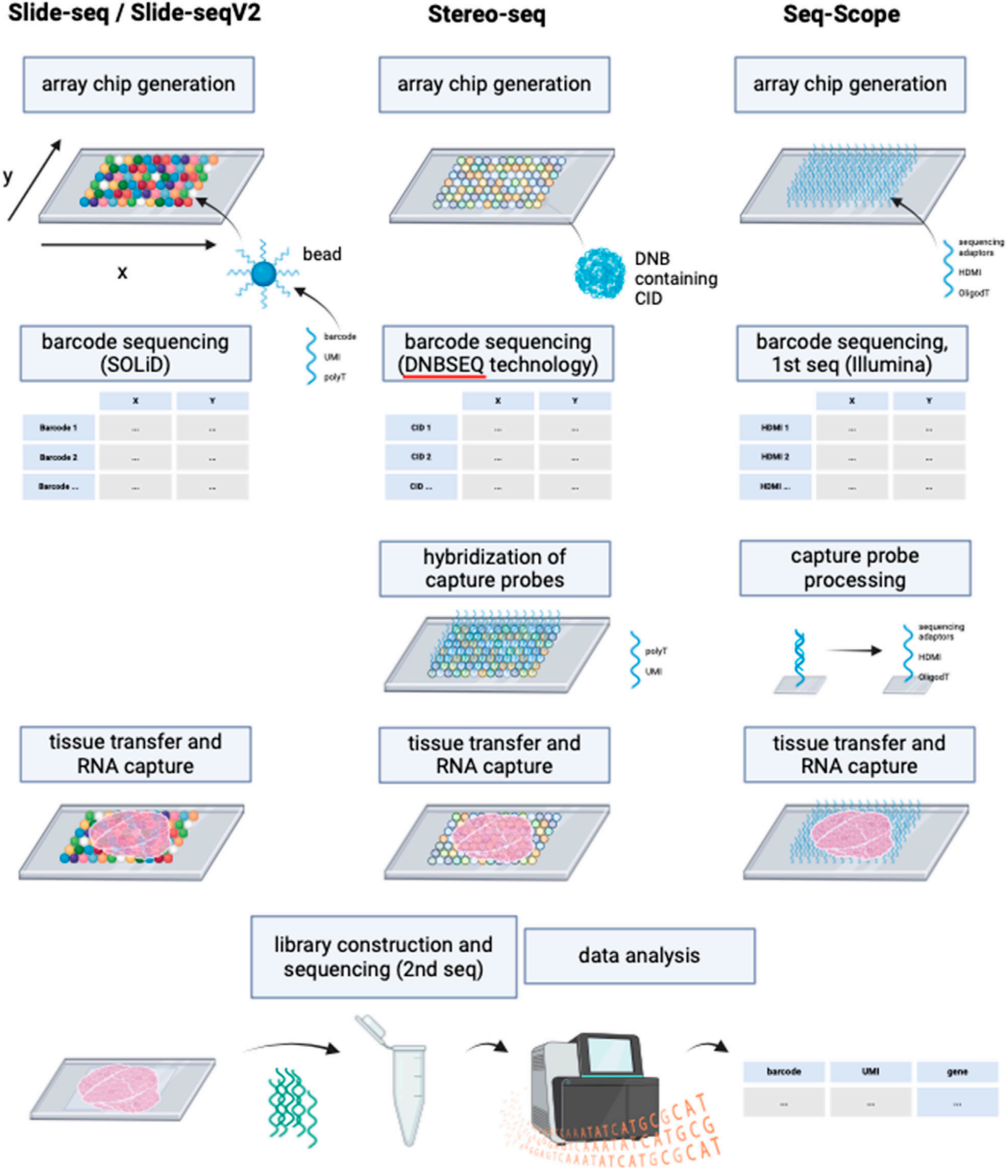
Schematic representation of the working principles of Slide-seq/Slide-seqV2, Stereo-seq and Seq-Scope. Although deploying different technologies, these NGS-based methods with region capture share resembling steps. While Slide-seq/Slide-seqV2 uses DNA-barcoded beads for RNA capture from tissue, Stereo-seq utilizes DNA balls (DNB) containing the barcode called coordinate identity (CID). Hybridization of oligonucleotides to the DNBs is then required for the following RNA capture from tissue. Seq-scope by contrast generates an array chip by high-definition map coordinate identifier (HDMI)-containing oligonucleotide amplification on a flow cell surface. All three methods have in common the barcode sequencing step after array chip generation to obtain coordinates for the barcodes’ location. In Seq-scope, processing is a required step before tissue transfer, to reveal sequences for RNA capture that have been hidden by sequencing-by-synthesis during first seq. Tissue is transferred onto the slides to enable RNA capture, followed by library construction to prepare for NGS (called 2^nd^ seq in Seq-scope) and data analysis to infer RNA identity. Created with BioRender.com.

Slide-seq (Rodriques et al., 2019) and Slide-seqV2 (Stickels et al., 2021) use arrays with adjacently positioned barcoded beads. After mRNA capture from the sliced tissue on the barcoded beads, the library is amplified and sequenced via NGS. The location of the transcripts can then be inferred from the barcodes. Slide-seq achieves a spatial resolution of 10 μm, which is significantly higher than the resolution of VISIUM (55 µm) and even than the combined method of VISIUM and ExM (20 µm). An optimized version of Slide-seq, Slide-seqV2 yields a nearly 10-fold increase in transcript detection per bead compared to the original version (Stickels et al., 2021) (Figure 6; Table 1). Thus, it becomes compelling to consider the implementation of ExM protocols within the framework of Slide-seq methodology, with the potential to surpass existing resolution limitations and enhance the efficacy of RNA detection.

**TABLE 1:** Comparison of VISIUM, Slide-seqVs, stereo-seq and seq-scope.

Method	VISIUM	Slide-seqV2	Stereo-seq	Seq-scope
Spot size, spatial resolution	55 µm	10 µm	0.22 µm	<1 µm
Center-to-center distance	100 µm	10 µm	0.5 µm	0.5 µm
Spots per 100 μm^2^	0.01	1	400	100
Capture area	42.25 mm^2^	7 mm^2^	200 mm^2^	0.02 mm^2^
Capture efficiency (number of transcripts captured)	∼500 transcripts per spot (diameter: 55 µm)	∼550 transcripts per bead (diameter: 10 µm)	∼1,450 transcripts per area with a diameter 10 µm	∼850 transcripts per area with a diameter 10 µm

Similarly, the potential application of ExM on spatiotemporal enhanced resolution omics sequencing (Stereo-seq) could be explored. Developed to enable precise spatial transcriptomics on larger tissue sections e.g., for embryological research using a whole mouse embryo, Stereo-seq chips offer a field of view from 1 cm × 1 cm up to 13.2 cm x 13.x cm and provide nanoscale resolution of transcripts (Chen et al., 2022). The method deploys DNA nanoballs (DNBs) containing barcodes and capture probes, situated in the spots of the silicon chip. RNAs are captured onto the DNBs, followed by library construction and NGS (Chen et al., 2022).

Another highly interesting method is Seq-Scope which is based on two rounds of sequencing (Cho et al., 2021). First-Seq reads the barcode sequence of clusters of randomly barcoded oligonucleotides which are fixed on a solid surface, thus mapping the barcodes to their location. The oligonucleotides are then processed to enable RNA capture and 2nd-Seq takes place to return a dataset of the mRNA reads which can be traced back to their location by the barcode sequence. Seq-Scope can detect cellular compartments based on the detection of mitochondrial transcripts and on the amount of intronic sequences in the RNAs, which can indicate unspliced, nuclear RNA.

The major differences between the NGS-based methods with region capture lies in their resolution capacity based on spot sizes, center-to-center distances on the microarray and the total field of view, i.e., the size of the tissue that can be studied. Theoretical compatibility of these methods with ExM requires proper fixation and permeabilization of the tissue, ensuring that spatial relationships can be maintained during expansion and RNA accessibility for subsequent RNA capture. Although these methods’ chemistries might be compatible with ExM in theory, further research is needed to validate the combination with ExM experimentally. The integration of ExM with these methods holds the promise of surpassing existing resolution limitations and enhancing RNA detection efficacy, warranting further research and development (Rodriques et al., 2019; Cho et al., 2021; Stickels et al., 2021; Chen et al., 2022).

## 3 Applications

Despite the high complexity of transcriptomics methods, they have been applied to tackle a great number of research questions in recent years. Their ability to enable the study of cell identity, function and interaction in tissues renders them very valuable. Recently, the development of transcriptomic methods has been advanced to maintain spatial information of the registered gene expression, which can further be enhanced by combining spatial transcriptomics methods with the ExM technology. Until today, these new methods have not been widely-used yet but they have great potential to be put into practice, encouraging discoveries to be made in a variety of different research fields.

Here, we want to give some examples of how spatial transcriptomics in general has advanced our knowledge in some research fields over the last years.

### 3.1 Identification of cell types

Studying the gene expression patterns received from transcriptomic data enables more precise characterization of cells, the detection of rare cells, as well as cellular subpopulations (Ke et al., 2016). Moreover, cell atlases can be constructed, providing information about gene expression patterns in, e.g., human or mouse cells (Zhuang, 2021; Moffitt et al., 2022; Vu et al., 2022). For instance, whole mouse brain atlases were created from spatial transcriptomic analysis by MERFISH and single-cell RNA sequencing (scRNA-seq) of approximately 7-8 million cells (Yao et al., 2023; Zhang et al., 2023). Similarly, a human cell atlas of the middle and superior temporal gyrus has been reported (Fang et al., 2022).

### 3.2 Examining the spatial distribution of cell types in a specific tissue

Cells can be grouped into clusters, thus providing information about regional distribution of cells as well as cell activity and state. Such studies have especially been carried out in various regions of the brain, e.g., the primary motor cortex, the visual cortex or the hippocampus, but they have also been used to study other tissues such as the embryonic heart and neural crest (Zhuang, 2021).

### 3.3 Insights into cellular function based on the intracellular spatial distribution of transcripts

On a single-cell level, valuable information can be obtained, e.g., about the underlying mechanisms of cell motility, cell function, cell survival, lineage and development (Zhuang, 2021; Tian et al., 2022; Vu et al., 2022; Williams et al., 2022). Spatial location of RNAs can efficiently be detected in the subcellular compartments using transcriptomic methods, and as RNA location serves as a post-transcriptional regulation mechanism, the data obtained enables insights into the cellular processes and activities taking place (Zhuang, 2021). For instance, differences in the mRNA types present in the neuronal soma, apical and basal dendrites have been observed by the use of targeted ExSeq, with the expression of structural genes enriched in the dendrites and spines (Alon et al., 2021). Furthermore, neuronal circuits can be studied in the brain by high-throughput transcriptomic methods (X. Chen et al., 2019). Moreover, transcriptomic methods can be used to identify somatic mutations in order to determine cell lineage (Frieda et al., 2017). Transcriptomics techniques such as MERFISH have also been used to target DNA, thus enabling the study of the genome. For instance, the chromatin can be imaged in a 3-dimensional manner (Su et al., 2020). Su et al. targeted >1,000 genetic loci and nascent RNAs, and nuclear structures simultaneously to obtain spatial information on the chromatin interactions and the transcriptional process (Su et al., 2020). Such experiments have the potential to further reveal the role of chromatin organization, gene expression and gene regulation in governing cell functions (Ke et al., 2016).

### 3.4 Unveiling cellular and intercellular changes in the development of diseases

Spatial transcriptomics methods offer a powerful toolset for uncovering cellular and intercellular changes critical to understanding the development and progression of diseases. By providing spatially resolved gene expression data within tissue samples, these methodologies enable researchers to explore how specific genes are expressed and regulated in different cell types and their microenvironment (Cheng et al., 2023). This spatial context is invaluable for dissecting complex disease mechanisms, such as tumor heterogeneity, immune cell infiltration, and tissue remodeling (Tian et al., 2022; Williams et al., 2022). Additionally, spatial transcriptomics facilitates the identification of spatially localized molecular signatures associated with disease states, enabling the discovery of novel biomarkers and therapeutic targets. Furthermore, these methods can elucidate the spatial organization of cellular interactions and communication networks within diseased tissues, shedding light on the underlying biological processes driving disease progression (Cheng et al., 2023). Overall, the application of spatial transcriptomics in disease research holds potential for advancing our understanding of disease pathogenesis and for guiding the development of more effective diagnostic and therapeutic strategies tailored to individual patients.

Spatial transcriptomics methods have been utilized to gain insights into diverse pathomechanisms of diseases, as Table 2 shows. However, it can be observed that the adoption of many new methodologies is constrained to their specific research settings they have been developed in, whereas those achieving wider outreach frequently leverage commercial platforms (e.g., 10x Genomics VISIUM and MERFISH). Unfortunately, spatial transcriptomics methods leveraging ExM also have not yet spread beyond the institutes they have been developed in. Table 3 summarizes cell types and tissues that have previously been validated for the methods mentioned in this article.

**TABLE 2:** Selection of Studies applying spatial transcriptomics technology.

Study	Disease studied	Tissue/cell type used	spatial transcriptomics method used
Al-Holou et al. (2023)	therapy resistance and recurrence of glioblastoma	human biopsies	10x Genomics VISIUM
Lucas et al. (2023)	intratumor heterogeinity of meningiomas to identify targeted therapy	human biopsies	10x Genomics VISIUM
Van de Velde et al. (2021)	role of immune cells in neuroblastoma formation	tumors from OT-II mice	10x Genomics VISIUM
Niño et al. (2022)	tumor microenvironment (TME) of oral squamous cell carcinoma and colorectal cancer	human tumor tissue	adapted VISIUM method
Zhu et al. (2022)	lung adenocarcinoma: cell atlas and TME	human biopsies	10x Genomics VISIUM
Stur et al. (2022)	serous ovarian carcinoma and response to chemotherapy	High-grade serous ovarian carcinoma biopsy	10x Genomics VISIUM
Misra et al. (2021)	Regeneration of the neonatal heart after scarring	neonatal hearts after apical resection surgery in mice	10x Genomics VISIUM
Hendricks et al. (2022)	Trem2-expressing macrophages in non-alcoholic steatohepatitis (NASH)	mouse liver tissue	10x Genomics VISIUM
Carpenter et al. (2023)	microenvironment of the adult human pancreas and pancreatic intraepithelial neoplasia lesions	pancreatic tissue from brain dead donors	10x Genomics VISIUM
Vickovic et al. (2022)	rheumatoid arthritis	synovial biopsies	spatial transcriptomics Ståhl et al
Carlberg et al. (2019)	rheumatoid arthritis, spondyloarthritis	synovial biopsies	spatial transcriptomics Ståhl et al
Gregory et al. (2020)	Transcription dysregulation in amyotrophic lateral sclerosis	*post mortem* brain tissue	spatial transcriptomics Ståhl et al
Maniatis et al. (2019)	amyotrophic lateral sclerosis	lumbal spinal cord tissue section from a mouse model	spatial transcriptomics Ståhl et al
Chen et al. (2024)	nucleus pulposus progenitor cells	mouse intervertebral discs	10x Genomics VISIUM
Sounart et al. (2023)	COVID-19	human *postmortem* lung tissue	10x Genomics VISIUM
Asp et al. (2019)	Human heart development, cell atlas	Human developmental heart tissue	spatial transcriptomics Stahl et al
Du et al. (2023)	Transcriptional profile of epicardial cells	mouse heart tissue	10x Genomics VISIUM
Androvic et al. (2023)	brain injury	murine brain tissue	MERFISH with electron microscopy
Park et al. (2023)	fate determination of neoblast cells	Schmidtea mediterranea	MERFISH
Lu et al. (2021)	hematopoiesis in fetal liver	murine wild type and Tet2^−/−^ fetal liver tissue	MERFISH
Cadinu et al. (2023)	colitis	colon tissue from a mouse colitis model	MERFISH
Boussaty et al. (2023)	age-related hearing loss	aging mouse cochlea	MERFISH
Shi et al. (2023)	sickle cell anemia	sickle cell anemia mouse liver	MERFISH
Johnston et al. (2023)	Alzheimer’s disease	mouse brain sections	MERFISH

**TABLE 3:** Cell type and tissue compatibility of spatial transcriptomics methods with and without ExM technology.

	Spatial transcriptomics method	Cell types/tissues validated/tested for this method in the original reports
without ExM	FISSEQ (Lee et al., 2015)	• various cultured cell types (e.g. HeLa, 293 A, COS1, U-2 OS, iPSC, primary fibroblasts, bipolar neurons) • tissue sections (frozen and formalin-fixed and paraffin-embedded (FFPE) tissue sections) • iPS-cell derived organoids • whole mount Drosophila embryos
	MERFISH	• Human primary fibroblast cells (IMR90) (Chen et al., 2015) • Human osteosarcoma cells (U-2 OS) (Moffitt et al., 2016; Wang et al., 2019) • Mouse brain tissue (Moffitt et al., 2016; Moffitt et al., 2018; Zhang et al., 2021; Osterhout et al., 2022; Allen et al., 2023) • Mouse retinal tissue (Choi et al., 2023) • Human brain tissue (middle and superior temporal gyrus) (Fang et al., 2022) • Mouse fetal liver tissue (Lu et al., 2021) • Mouse liver tissue (Shi et al., 2023) • Neoblasts form Schmidtea mediterranea (Park et al., 2023) • Mouse intestinal tissue (Cadinu et al., 2023) • HEK293T cells (Barbash et al., 2019) • MCF10a cells (Foreman et al., 2020)
	VISIUM (Datasets - 10x Genomics, n.d.)	• various tissues from mice (brain, lung, kidney, embryo) • various human tissues (brain, spinal cord, lung, kidney, colon, breast, ovary, prostrate, lymph node)
with ExM	ExSeq (Alon et al., 2021)	• Mouse hippocampus tissue (targeted and untargeted approach) • Mouse visual cortex (targeted approach) • human breast cancer metastasis in liver tissue (targeted approach) • C. elegans (untargeted approach) • Drosophila embryos (untargeted approach) • HeLa cells (untargeted approach)
	MERFISH with ExM	• cultured human osteosarcoma cells (U-2 OS cells) (Wang et al., 2018; Xia, Fan, et al., 2019)
	VISIUM with ExM (Ex-ST)	• mouse brain tissue (Fan et al., 2023)

## 4 Strengths and limitations

Although all spatial transcriptomics methods have the ability to produce data on the spatial gene expression in cells and tissues, there are differences in the efficacy and applicability between ISS, ISH methods and NGS-based methods with region capture (Table 4).

**TABLE 4:** Comparison of ISS methods, ISH methods and NGS-based sequencing with region capture.

		ISS methods		ISH methods		NGS sequencing with region capture	
		FISSEQ	ExSeq	MERFISH	MERFISH with ExM	VISIUM	Ex-ST
Efficacy	RNA detection	detection of ca. 8,700 genes from ca. 150.000 reads <1% detection efficiency lower abundance transcripts might be missed	detection of ca. 3,000 genes within 10 microscope volumes ca. ∼ 62% detection efficiency in comparison with HCRv3.0-ExFISH lower abundance transcripts detectable	detection of ca. 1,000 genes ∼95% detection efficiency compared to smFISH lower abundance transcripts detectable	detection of up to 10.000 genes near 100% detection efficiency compared to smFISH lower abundance transcripts detectable	ca. 230 genes captured (ca. 500 RNAs per spot) ∼7% detection efficiency compared to smFISH	ca. 430 genes per spot captured (ca. 680 RNAs per spot)
	detection accuracy	99,36% base calling efficiency 90,6% correct assignment of amplicon to gene	not specified	80% RNA detection sensitivity low misidentification rate because of error-robust barcoding	80% RNA detection sensitivity only 4% misidentification rate because of error-robust barcoding	spot occupancy of a single cell type ∼50%	spot occupancy of a single cell type ∼80% reduced signal integration from other cells in one spot
	possibilities of upscaling	application of ExM technology or other superresolution methods	usage of a greater expansion factor for ExM application of other superresolution methods	application of ExM technology or other superresolution methods introduction of more hybridization rounds	usage of a greater expansion factor for ExM application of other superresolution methods introduction of more hybridization rounds	application of ExM technology or other superresolution methods	usage of a greater expansion factor for ExM application of other superresolution methods
	limitations	tissue integrity affected by multiplexing RNA read length: 5–30 bp	tissue integrity affected by multiplexing RNA read length: 5–30 bp	tissue integrity affected by multiplexing detection of short RNA is less accurate	tissue integrity affected by multiplexing short RNAs of lengths <500 nt not detectable	RNA from diverse cells in one spot (1–10 cells)	RNA from diverse cells in one spot
	error handling	-	additional *ex-situ* NGS on *in situ* sequenced transcripts obtaining longer read lengths to enable better RNA identification	accurate RNA detection despite mishybridization possible through error detection and correction by robust barcoding	accurate RNA detection despite mishybridization possible through error detection and correction by robust barcoding	-	-
Resolution	resolution capacity and RNA localization	single-cell and subcellular resolution amplicon size: 200–400 nm	single-cell and enhanced subcellular resolution amplicon size 50–100 nm	single-cell and subcellular resolution	single-cell and enhanced subcellular resolution	not single-cell resolution low resolution (55 µm) limited by the microarray design	not single-cell resolution improved spatial resolution of 20 µm compared to standard VISIUM
Applicability	cells/tissue size	single-cells and small tissues (e.g., *Drosophila* embryo)	single-cells and small tissues	single-cells and small tissues	cultured cells	large tissues cryosectioned or FFPE tissue size: 6.5 × 6.5 mm	tissues
	targetedness	targeted and untargeted	targeted and untargeted	only targeted	only targeted	targeted and untargeted	targeted and untargeted
Feasibility	cost	costly, specialized equipment needed	costly, specialized equipment needed	costly, specialized equipment needed	costly, specialized equipment needed	less costly than ISS and ISH methods, NGS machinery needed	less costly than ISS and ISH methods, NGS machinery needed
	duration	ranges from several days to weeks	longer than FISSEQ	ranges from several days to weeks	longer than MERFISH	a few days	longer duration than VISIUM (ca. +3 days)
	commercialized platform	-	-	MERSCOPE by Vizgen	-	VISIUM by 10X Genomics	-

The methods mentioned in this article are compared by their efficiency, resolution, applicability and feasibility. Ca. = circa, bp = base pairs, nt = nucleotides. (Lee et al., 2014; Chen et al., 2015; Lee et al., 2015; Ståhl et al., 2016; Wang et al., 2018; Xia, Fan, et al., 2019; Alon et al., 2021; Eng, 2021; Gracia Villacampa et al., 2021; Zhuang, 2021; Moses and Pachter, 2022; Fan et al., 2023).

Generally, a distinction must be made between targeted and untargeted approaches. While a huge advantage of untargeted approaches, such as ISS and NGS-based methods with region capture, is that they enable the unbiased discovery of genes in a particular region, targeted approaches, such as MERFISH, focus on preselected genes, making knowledge about the targeted RNAs prerequisite for the experimental design. A disadvantage of untargeted approaches is that they typically suffer from lower detection efficiencies (Moses and Pachter, 2022). These detection efficiencies are usually obtained by comparison of the method’s performance to the performance of non-barcoded smFISH on defined marker genes (Moses and Pachter, 2022). For instance, detection efficiencies of FISSEQ have been estimated to account for <1% partly due to attributed limitations associated with the optical crowding of amplicons, reverse transcription and sequencing-by-ligation procedures (Eng, 2021; Zhang et al., 2021; Moses and Pachter, 2022)). Similarly, the detection efficiency of VISIUM is low (7%) (Ståhl et al., 2016) in contrast to the detection efficiencies of ISH methods (∼95%) (Moses and Pachter, 2022). However, ISS and ISH methods excel similarly when compared by detection accuracy: Whereas FISSEQ assigns ∼90% of amplicons to the correct RNA (Lee et al., 2014), MERFISH and MERFISH combined with ExM achieve a correct identification of RNAs of 80% (Chen et al., 2015; Xia, Fan, et al., 2019). The latter is achieved on the one hand because FISH probe binding is highly sensitive and moreover, not every base needs to be detected as RNAs are identified using a number of specific probes per RNA. On the other hand, these methods employ error detection and correction through robust barcoding.

Concerning imaging, various imaging modalities, including widefield fluorescence microscopy, confocal microscopy, and light sheet microscopy, offer distinct advantages and limitations in spatial transcriptomics studies. Each imaging modality provides unique benefits and constraints that must be carefully considered. Widefield microscopy provides fast imaging with a wide field of view but lacks depth resolution, whereas confocal microscopy offers improved optical sectioning at the expense of imaging speed and cost. Lightsheet microscopy combines superior optical sectioning with high-speed imaging, making it well-suited for thick, 3D samples, albeit with greater complexity and initial investment.

A limiting factor of spatial transcriptomics methods is the resolution obtainable. A challenge arises when the amount of genes to be interrogated is increased, as it leads to elevated levels of optical crowding, thereby imposing constraints on image resolution. A solution to increasing resolution power beyond the resolution limit of the microscope and reducing optical crowding while at the same time allowing for more genes to be interrogated, is the application of the super resolution technique ExM. There are other super resolution technologies that could also help alleviate the problem. These techniques include single-molecule localization microscopy (SMLM), stimulated emission depletion (STED), DNA-point accumulation for imaging in nanoscale topography (DNA-PAINT) techniques. However, as the application of these techniques on spatial transcriptomics is out of the scope of this review, we want to refer to a highly interesting review exploring these super resolution techniques: Schermelleh et al., 2019; Schermelleh et al., 2019).

Furthermore, ISS and ISH methods obtain higher resolution than NGS-based methods with region capture and are able to reveal subcellular localization of RNAs. The implementation of ExM technology on these methods further increases RNA detection (Xia, Fan, et al., 2019; Alon et al., 2021). An interesting approach to studying subcellular compartmentalization of RNA has been reported: Visualization of subcellular structures (e.g., nucleus, ER) by antibody-labeling allows for conclusions to be drawn about the spatial relation of the detected RNAs to those cellular compartments (Chen et al., 2015; Xia, Fan, et al., 2019; Alon et al., 2021). Also for NGS-based methods involving region capture, the application of ExM is a very promising way of increasing resolution power and RNA detection. Overall, NGS-based methods with region capture have the lowest resolution of the three classes of methods described in this review. However, they are designed to enable gene expression analysis across a whole tissue section without requiring highly specialized imaging equipment. Their accuracy is also limited by the lateral diffusion of RNAs during transfer from tissue to the array (Eng, 2021). Many NGS-based methods with region capture have emerged lately with by far better resolution capacity than VISIUM to approach single-cell resolution (e.g., Slide-seqV2, Stereo-seq, Seq-Scope). However, these methods have not yet been combined with ExM technology. Considering that Ex-ST was able to significantly increase the resolution of standard VISIUM, but still did not attain single-cell resolution, the application of ExM on one of these newer NGS-based sequencing methods with region capture is very promising.

Notably, RNA length is a limiting factor to spatial transcriptomics methods. Hence, ISS methods employing sequencing-by-ligation are restricted in the number of bases that can be called due to challenges such as optical degradation during imaging and enzymatic reactions that fail to reach completion, leading to “phasing” effects where signal dependency on preceding cycles occurs (Alon et al., 2021). These methods typically generate only 30-base long reads of RNA transcripts (Lee et al., 2014; Alon et al., 2021). Shorter reads are more likely to match to multiple RNA sequences in the transcriptome, possibly leading to misidentification of transcripts. Also, by relying on shorter reads, the detection of variations within RNA sequences, such as single-nucleotide polymorphisms (SNPs) or splicing variants might prove challenging (Alon et al., 2021). Alon et al. tackle this problem by applying *ex-situ* NGS on the RNA transcripts previously sequenced *in situ* which are identified by UMIs (Alon et al., 2021). The same issue of limited RNA length and variant detection applies to VISIUM. Recently, an adapted VISIUM method named “Spatial Isoform Transcriptomics” has been published tackling this issue (Lebrigand et al., 2023). In contrast, FISH probes need to be specifically designed to target SNPs and splice isoforms in ISH techniques and thus require extra effort. However, ISH methods encounter challenges when dealing with short RNA transcripts. For example, RNAs of lengths <500 nt cannot be detected in MERFISH with ExM (Xia, Fan, et al., 2019). However, this could be improved to detect RNA lengths of 100–200 nt by branched DNA amplification and intelligent overlapping encoding probe design (Xia, Babock, et al., 2019).

In general, *in situ* multiplexing on the same tissue, inherent to ISS and ISH approaches, might negatively affect cellular structure and could lead to errors (Vu et al., 2022). For instance, fluorescence signal intensity decreases during MERFISH experiments as a sign of tissue degradation (Chen et al., 2015). Thus, ISH methods usually apply error-robust barcoding to infer accurate RNA detection despite eventual errors, e.g., by mishybridizations or tissue degradation. Also, ISS and ISH methods are usually carried out on smaller tissues or cultured cells, because the cost and duration increase the larger the tissue to be studied is. This also serves as a downside of the application of ExM on these methods, as expanding tissues physically increases tissue area. Overall, referring to the duration of the total spatial transcriptomics experiment, the execution of ExM protocols requires additional time and thus extends total duration. However, the application of ExM could spare time in ISH methods: When considering to upscale a MERFISH experiment, the alternative to the application of ExM would be the addition of supplementary hybridization cycles which is very time-consuming and might take even longer in total then the performance of ExM on the tissue (Xia, Fan, et al., 2019). Also, the addition of hybridization cycles comes at the cost of increased molecular crowding (Chen et al., 2015).

Another benefit of applying ExM for spatial transcriptomics is the reduction of background fluorescence of tissues (Xia, Fan, et al., 2019). This is achieved because the cytoplasm gets “diluted” during expansion and it is particularly of advantage in tissues that exhibit high levels of autofluorescence.

While the application of ExM offers numerous advantages, it is essential to consider the potential challenges and limitations introduced by this technique. The ExM processing steps, which involve the enzymatic digestion of tissue proteins and the subsequent physical expansion of the sample, may lead to the loss of RNA molecules. This is particularly problematic for low abundance transcripts, as their detection might be further compromised by the dilution effect during expansion. Additionally, the reproducibility of experiments can be affected by variations in the expansion process, such as inconsistencies in the degree of expansion and potential differential diffusion of RNA molecules. It is crucial to optimize and standardize the ExM protocols to minimize these effects and ensure reliable and reproducible spatial transcriptomics data. Strategies such as the use of stabilizing agents or improved anchoring techniques for RNA molecules could mitigate these issues and enhance the overall robustness of combined ExM and spatial transcriptomics methodologies.

Thus, the application of ExM on all three classes of spatial transcriptomics methods (ISS, ISH and NGS-based sequencing with region capture) proves very promising and exciting. However, there is still room for improvement of these single methods presented here which remains subject to further research. Additionally, testing the performance of the application of ExM on other methods bares great potential and should be encouraged, especially on NGS-based methods with region capture whose implementation usually proves to be simpler into a standard laboratory.

**BOX - Benefits of ExM application on spatial transcriptomics experiments.**

• enhanced resolution power by optical crowding reduction of RNA transcripts
• more exact RNA localization to their subcellular compartment
• increased capacity for gene interrogation, detection and identification
• reduction of background autofluorescence

## 5 Conclusion

In summary, this review provides an exploration into the landscape of spatial transcriptomics methods, shedding light on their classification and enhancement through the innovative application of Expansion Microscopy (ExM).

On the one hand, the category of *in situ* sequencing methods, exemplified by Fluorescent *In Situ* Sequencing (FISSEQ) and Expansion Sequencing (ExSeq), directly sequences RNA in the cell and tissue. FISSEQ, introduced in 2014, employs reverse transcription (RT), cDNA circularization, and rolling circle amplification (RCA) to generate amplicons for library preparation. Subsequently, *in situ* SOLiD sequencing utilizes an off-set addition of primers and 5-nucleotide probes to determine RNA sequences. This ISS approach can be significantly advanced by combination with ExM, a method which has been developed in 2021 by Alon et al.

On the other hand, Fluorescence *In Situ* Hybridization (FISH) methods are based on the delivery of FISH probes into the cells for hybridization to a target sequence. MERFISH enables the simultaneous visualization of numerous RNA targets by the assignment of a binary barcode to the targeted RNAs and the encoding of the barcode by smart encoding probe and readout probe design for each targeted RNA. The readout of the barcode during imaging cycles is obtained by whether a fluorescence signal can be observed in a specific spot or not and thus, depending on the barcode obtained the RNA identity can be determined. Furthermore, the application of expansion microscopy on MERFISH shows that enhancements can be made in terms of the amount of RNA detected per cell by reducing optical crowding and thereby increasing resolution power.

Next, we elaborated on NGS methods with region capture, exemplified by the VISIUM platform. Herein, RNAs from tissue sections are transferred onto a microarray which contains the capture probes binding the RNA. These capture probes contain specific barcodes to encode the initial location of the RNA on the microarray. Following RNA preparation, Illumina sequencing is performed to obtain the RNA sequences.

Moving beyond the techniques and procedures, we highlighted the broader implications of transcriptomics in biology and medicine. Transcriptomics methods and especially spatial transcriptomics methods offer valuable insight into the spatial organization of gene expression in cells within tissues, holding great promise for advancing diagnostic and therapeutic approaches. By understanding the complex molecular mechanism of diseases, novel biomarkers and therapeutic targets could be uncovered, fostering a more nuanced and personalized approach to medicine. However, the clinical implementation of spatial transcriptomics methods remains a distant goal, necessitating further optimization of methodologies, commercialization to enhance usability in clinical settings, and the establishment of associations between gene expression profiles and diseases to establish diagnostic standards.

Finally, we addressed the benefits and limitations inherent in the methodologies mentioned. While ISS methods compared to ISH technologies allow for an untargeted approach to studying gene expression, they generally yield a lower RNA detection efficacy than ISH technologies. In contrast to these both, NGS-based sequencing with region capture is mainly limited by the microarrays used to capture the RNAs, thus leading to a significantly lower RNA detection rate than ISS and ISH methods by not achieving single cell resolution. One advantage of NGS-based transcriptomics methods is that it also enables an untargeted approach to transcriptomics. Inherent to all of these methods are challenges such as sample heterogeneity, data analysis complexity, and cost considerations which underscore the need for continued innovation.

While spatial transcriptomics has undeniably revolutionized our ability to study cellular organization, it has driven the development of a great number of innovative transcriptomics methods such as ISS, ISH and NGS-based technologies.These techniques not only refine our understanding of cellular processes but also open up new possibilities for breakthroughs in various research domains. In this review, we showed that the combination of transcriptomic protocols with ExM has been able to extend the capabilities of these techniques by increasing RNA detection and improving resolution power. The comparison of ISS with ISH and NGS-based methods showed that for each technology, there are challenges to overcome and the researcher should choose the method that fits most their experiment design and expected results. The study of spatial transcriptomics is ongoing and we look forward to all the discoveries to be made.

## Figures and Tables

**Table:** 

**APS**	ammonium persulfate
**DNA-PAINT**	DNA-point accumulation for imaging in nanoscale topography
**DNB**	DNA nanoball
**Ex-ST**	Expansion spatial transcriptomics
**ExM**	Expansion Microscopy
**ExPath**	Expansion Pathology
**ExSEQ**	Expansion sequencing
**FFPE**	formalin-fixed paraffin embedded
**FISH**	Fluorescence *in situ* hybridization
**FISSEQ**	Fluorescent *in situ* RNA sequencing
**HE**	hematoxylin and eosin
**ISH**	*in situ* hybridization
**ISS**	*in situ* sequencing
**LNA**	locked nucleic acid probes
**MERFISH**	Multiplexed error-robust fluorescence *in situ* hybridization
**NASH**	non-alcoholic steatohepatitis
**NGS**	next-generation sequencing
**PBS**	phosphate-buffered saline
**PCR**	polymerase chain reaction
**RCA**	rolling circle amplification
**RT**	reverse transcription
**RT-qPCR**	Real Time Quantitative PCR
**scRNA-seq**	single-cell RNA sequencing
**SDS**	sodium dodecyl sulfate
**smFISH**	single-molecule FISH
**SSC**	saline-sodium citrate
**SMLM**	single-molecule localization microscopy
**SOLiD**	sequencing by oligonucleotide ligation and detection
**STARmap**	spatially resolved transcript amplicon readout mapping
**STED**	stimulated emission depletion
**Stereo-seq**	spatiotemporal enhanced resolution omics sequencing
**TEMED**	tetramethylethylenediamine
**TME**	tumor microenvironment
**TSO**	template switch oligo
**UMI**	unique molecular identifier

## References

[B1] Al-Holou W. N. Wang H. Ravikumar V. Shankar S. Oneka M. Fehmi Z. (2023). Subclonal evolution and expansion of spatially distinct THY1-positive cells is associated with recurrence in glioblastoma. Neoplasia (New York, N.Y.) 36, 100872. 10.1016/J.NEO.2022.100872 36621024 PMC9841165

[B2] Allen W. E. Blosser T. R. Sullivan Z. A. Dulac C. Zhuang X. (2023). Molecular and spatial signatures of mouse brain aging at single-cell resolution. Cell. 186 (1), 194–208.e18. 10.1016/J.CELL.2022.12.010 36580914 PMC10024607

[B3] Alon S. Goodwin D. R. Sinha A. Wassie A. T. Chen F. Daugharthy E. R. (2021). Expansion sequencing: spatially precise *in situ* transcriptomics in intact biological systems. Sci. (New York, N.Y.) 371 (6528), eaax2656. 10.1126/SCIENCE.AAX2656 PMC7900882 33509999

[B4] Androvic P. Schifferer M. Perez Anderson K. Cantuti-Castelvetri L. Jiang H. Ji H. (2023). Spatial Transcriptomics-correlated Electron Microscopy maps transcriptional and ultrastructural responses to brain injury. Nat. Commun. 14 (1), 4115. 10.1038/S41467-023-39447-9 37433806 PMC10336148

[B5] Asp M. Giacomello S. Larsson L. Wu C. Fürth D. Qian X. (2019). A spatiotemporal organ-wide gene expression and cell atlas of the developing human heart. Cell. 179 (7), 1647–1660. 10.1016/J.CELL.2019.11.025 31835037

[B6] ATDBio (2023). Next generation sequencing. Available at: https://atdbio.com/nucleic-acids-book/Next-generation-sequencing#Sequencing-by-ligation-SOLiD.

[B7] Barbash S. Persson T. Lorenzen E. Kazmi M. A. Huber T. Sakmar T. P. (2019). Detection of concordance between transcriptional levels of GPCRs and receptor-activity-modifying proteins. IScience 11, 366–374. 10.1016/J.ISCI.2018.12.024 30660104 PMC6354700

[B8] Bayani J. Squire J. A. (2004). Fluorescence *in situ* hybridization (FISH). Curr. Protoc. Cell. Biol. 22, Unit 22.4. 10.1002/0471143030.CB2204S23 18228455

[B9] Boussaty E. C. Tedeschi N. Novotny M. Ninoyu Y. Du E. Draf C. (2023). Cochlear transcriptome analysis of an outbred mouse population (CFW). BioRxiv Prepr. Serv. Biol. 10.1101/2023.02.15.528661 PMC10716316 38094513

[B10] Bucur O. Fu F. Calderon M. Mylvaganam G. H. Ly N. L. Day J. (2020). Nanoscale imaging of clinical specimens using conventional and rapid expansion pathology. Nat. Protoc. 15 (5), 1649–1672. 10.1038/S41596-020-0300-1 32238952 PMC7490752

[B11] Bucur O. Zhao Y. (2018). Nanoscale imaging of kidney glomeruli using expansion pathology. Front. Med. 5 (NOV), 322. 10.3389/FMED.2018.00322 PMC6259469 30519560

[B12] Cadinu P. Sivanathan K. N. Misra A. Xu R. J. Mangani D. Yang E. (2023). Charting the cellular biogeography in colitis reveals fibroblast trajectories and coordinated spatial remodeling. BioRxiv. 10.1101/2023.05.08.539701 PMC11017707 38569542

[B13] Carlberg K. Korotkova M. Larsson L. Catrina A. I. Ståhl P. L. Malmström V. (2019). Exploring inflammatory signatures in arthritic joint biopsies with Spatial Transcriptomics. Sci. Rep. 9 (1), 18975. 10.1038/S41598-019-55441-Y 31831833 PMC6908624

[B14] Carpenter E. S. Elhossiny A. M. Kadiyala P. Li J. McGue J. Griffith B. D. (2023). Analysis of donor pancreata defines the transcriptomic signature and microenvironment of early neoplastic lesions. Cancer Discov. 13 (6), 1324–1345. 10.1158/2159-8290.CD-23-0013 37021392 PMC10236159

[B15] Chen A. Liao S. Cheng M. Ma K. Wu L. Lai Y. (2022). Spatiotemporal transcriptomic atlas of mouse organogenesis using DNA nanoball-patterned arrays. Cell. 185, 1777–1792.e21. 10.1016/j.cell.2022.04.003 35512705

[B16] Chen J. McSwiggen D. Ünal E. (2018). Single molecule fluorescence *in situ* hybridization (smFISH) analysis in budding yeast vegetative growth and meiosis. J. Vis. Exp. JoVE 2018 (135), 57774. 10.3791/57774 PMC6101419 29889208

[B17] Chen K. H. Boettiger A. N. Moffitt J. R. Wang S. Zhuang X. (2015). RNA imaging. Spatially resolved, highly multiplexed RNA profiling in single cells. Sci. (New York, N.Y.) 348 (6233), aaa6090. 10.1126/SCIENCE.AAA6090 PMC4662681 25858977

[B18] Chen T. Y. You L. Hardillo J. A. U. Chien M. P. (2023). Spatial transcriptomic technologies. Cells 12 (16), 2042. 10.3390/CELLS12162042 37626852 PMC10453065

[B19] Chen X. Sun Y. C. Zhan H. Kebschull J. M. Fischer S. Matho K. (2019). High-throughput mapping of long-range neuronal projection using *in situ* sequencing. Cell. 179 (3), 772–786. 10.1016/J.CELL.2019.09.023 31626774 PMC7836778

[B20] Chen Y. Zhang L. Shi X. Han J. Chen J. Zhang X. (2024). Characterization of the nucleus pulposus progenitor cells via spatial transcriptomics. Adv. Sci. Weinheim, Baden-Wurttemberg, Ger. 11, e2303752. 10.1002/ADVS.202303752 PMC11095158 38311573

[B21] Cheng M. Jiang Y. Xu J. Mentis A. F. A. Wang S. Zheng H. (2023). Spatially resolved transcriptomics: a comprehensive review of their technological advances, applications, and challenges. J. Genet. Genomics 50 (9), 625–640. 10.1016/J.JGG.2023.03.011 36990426

[B22] Cho C. S. Xi J. Si Y. Park S. R. Hsu J. E. Kim M. (2021). Microscopic examination of spatial transcriptome using seq-scope. Cell. 184, 3559–3572.e22. 10.1016/J.CELL.2021.05.010 34115981 PMC8238917

[B23] Choi J. Li J. Ferdous S. Liang Q. Moffitt J. R. Chen R. (2023). Spatial organization of the mouse retina at single cell resolution by MERFISH. Nat. Commun. 14 (1), 4929. 10.1038/S41467-023-40674-3 37582959 PMC10427710

[B24] Datasets (2024). 10x Genomics. Available at: https://www.10xgenomics.com/datasets?query=&page=2&configure%5BhitsPerPage%5D=50&configure%5BmaxValuesPerFacet%5D=1000&refinementList%5Bproduct.name%5D%5B0%5D=Spatial%20Gene%20Expression.

[B25] Derrick C. J. Sánchez-Posada J. Hussein F. Tessadori F. Pollitt E. J. G. Savage A. M. (2022). Asymmetric Hapln1a drives regionalized cardiac ECM expansion and promotes heart morphogenesis in zebrafish development. Cardiovasc. Res. 118 (1), 226–240. 10.1093/cvr/cvab004 33616638 PMC8752364

[B26] Du J. Yuan X. Deng H. Huang R. Liu B. Xiong T. (2023). Single-cell and spatial heterogeneity landscapes of mature epicardial cells. J. Pharm. Analysis 13 (8), 894–907. 10.1016/J.JPHA.2023.07.011 PMC10499659 37719196

[B27] Eng C.-H. L. (2021). Plus ultra: genome-wide spatial transcriptomics with RNA seqFISH+.

[B28] Fan Y. Andrusivová Ž. Wu Y. Chai C. Larsson L. He M. (2023). Expansion spatial transcriptomics. Nat. Methods 2023 20 (8), 1179–1182. 10.1038/s41592-023-01911-1 PMC11078125 37349575

[B29] Fang R. Xia C. Close J. L. Zhang M. He J. Huang Z. (2022). Conservation and divergence of cortical cell organization in human and mouse revealed by MERFISH. Sci. (New York, N.Y.) 377 (6601), 56–62. 10.1126/SCIENCE.ABM1741 PMC9262715 35771910

[B30] Foreman R. Wollman R. (2020). Mammalian gene expression variability is explained by underlying cell state. Mol. Syst. Biol. 16 (2), 9146. 10.15252/MSB.20199146 PMC7011657 32043799

[B31] Frieda K. L. Linton J. M. Hormoz S. Choi J. Chow K. H. K. Singer Z. S. (2017). Synthetic recording and *in situ* readout of lineage information in single cells. Nature 541 (7635), 107–111. 10.1038/NATURE20777 27869821 PMC6487260

[B32] Galeano Niño J. L. Wu H. LaCourse K. D. Kempchinsky A. G. Baryiames A. Barber B. (2022). Effect of the intratumoral microbiota on spatial and cellular heterogeneity in cancer. Nature 611 (7937), 810–817. 10.1038/S41586-022-05435-0 36385528 PMC9684076

[B33] Garafutdinov R. R. Sakhabutdinova A. R. Gilvanov A. R. Chemeris A. V. (2021). Rolling circle amplification as a universal method for the analysis of a wide range of biological targets. Russ. J. Bioorg. Chem. 47 (6), 1172–1189. 10.1134/S1068162021060078 34931113 PMC8675116

[B34] Gracia Villacampa E. Larsson L. Mirzazadeh R. Kvastad L. Andersson A. Mollbrink A. (2021). Genome-wide spatial expression profiling in formalin-fixed tissues. Cell. Genomics 1 (3), 100065. 10.1016/J.XGEN.2021.100065 36776149 PMC9903805

[B35] Gregory J. M. McDade K. Livesey M. R. Croy I. Marion de Proce S. Aitman T. (2020). Spatial transcriptomics identifies spatially dysregulated expression of GRM3 and USP47 in amyotrophic lateral sclerosis. Neuropathology Appl. Neurobiol. 46 (5), 441–457. 10.1111/NAN.12597 31925813

[B36] Hendrikx T. Porsch F. Kiss M. G. Rajcic D. Papac-Miličević N. Hoebinger C. (2022). Soluble TREM2 levels reflect the recruitment and expansion of TREM2+ macrophages that localize to fibrotic areas and limit NASH. J. Hepatology 77 (5), 1373–1385. 10.1016/j.jhep.2022.06.004 35750138

[B37] Huang L. Fang L. Liu Q. Torshizi A. D. Wang K. (2022). Integrated analysis on transcriptome and behaviors defines HTT repeat-dependent network modules in Huntington’s disease. Genes. and Dis. 9 (2), 479–493. 10.1016/J.GENDIS.2021.05.004 PMC8843892 35224162

[B38] Hudson W. H. Sudmeier L. J. (2022). Localization of T cell clonotypes using the Visium spatial transcriptomics platform. Star. Protoc. 3 (2), 101391. 10.1016/J.XPRO.2022.101391 35707680 PMC9189629

[B39] Jia Q. Wu W. Wang Y. Alexander P. B. Sun C. Gong Z. (2018). Local mutational diversity drives intratumoral immune heterogeneity in non-small cell lung cancer. Nat. Commun. 9 (1), 5361. 10.1038/S41467-018-07767-W 30560866 PMC6299138

[B40] Johnston K. Berackey B. B. Tran K. M. Gelber A. Yu Z. MacGregor G. (2023). Single cell spatial transcriptomics reveals distinct patterns of dysregulation in non-neuronal and neuronal cells induced by the Trem2R47H Alzheimer’s risk gene mutation. Res. Square. 10.21203/RS.3.RS-3656139/V1 PMC11746152 39103533

[B41] Ke R. Mignardi M. Hauling T. Nilsson M. (2016). Fourth generation of next‐generation sequencing technologies: promise and consequences. Hum. Mutat. 37 (12), 1363–1367. 10.1002/HUMU.23051 27406789 PMC5111608

[B42] Ke R. Mignardi M. Pacureanu A. Svedlund J. Botling J. Wählby C. (2013). *In situ* sequencing for RNA analysis in preserved tissue and cells. Nat. Methods 10 (9), 857–860. 10.1038/nmeth.2563 23852452

[B43] Kolodziejczyk A. A. Kim J. K. Svensson V. Marioni J. C. Teichmann S. A. (2015). The technology and biology of single-cell RNA sequencing. Mol. Cell. 58 (4), 610–620. 10.1016/J.MOLCEL.2015.04.005 26000846

[B44] Lebrigand K. Bergenstråhlebergenstr˚bergenstråhle J. Thrane K. Mollbrink A. Meletis K. Barbry P. (2023). The spatial landscape of gene expression isoforms in tissue sections. Nucleic Acids Res. 51 (8), e47. 10.1093/NAR/GKAD169 36928528 PMC10164556

[B45] Lee J. H. Daugharthy E. R. Scheiman J. Kalhor R. Ferrante T. C. Terry R. (2015). Fluorescent *in situ* sequencing (FISSEQ) of RNA for gene expression profiling in intact cells and tissues. Nat. Protoc. 10 (3), 442–458. 10.1038/NPROT.2014.191 25675209 PMC4327781

[B46] Lee J. H. Daugharthy E. R. Scheiman J. Kalhor R. Yang J. L. Ferrante T. C. (2014). Highly multiplexed subcellular RNA sequencing *in situ* . Sci. (New York, N.Y.) 343 (6177), 1360–1363. 10.1126/SCIENCE.1250212 PMC4140943 24578530

[B47] Lewis S. M. Asselin-Labat M. L. Nguyen Q. Berthelet J. Tan X. Wimmer V. C. (2021). Spatial omics and multiplexed imaging to explore cancer biology. Nat. Methods 18 (9), 997–1012. 10.1038/s41592-021-01203-6 34341583

[B48] Lu Y. Liu M. Yang J. Weissman S. M. Pan X. Katz S. G. (2021). Spatial transcriptome profiling by MERFISH reveals fetal liver hematopoietic stem cell niche architecture. Cell. Discov. 7 (1), 47. 10.1038/S41421-021-00266-1 34183665 PMC8238952

[B49] Lubeck E. Cai L. (2012). Single-cell systems biology by super-resolution imaging and combinatorial labeling. Nat. Methods 9 (7), 743–748. 10.1038/nmeth.2069 22660740 PMC3418883

[B50] Lubeck E. Coskun A. F. Zhiyentayev T. Ahmad M. Cai L. (2014). Single cell *in situ* RNA profiling by sequential hybridization. Nat. Methods 11 (4), 360–361. 10.1038/NMETH.2892 24681720 PMC4085791

[B51] Lucas C.-H. Mirchia K. Seo K. Najem H. Chen W. Zakimi N. (2023). Spatial genomic, biochemical, and cellular mechanisms drive meningioma heterogeneity and evolution. Res. Square. 10.21203/RS.3.RS-2921804/V1 PMC11239374 38760638

[B52] Maniatis S. Äijö T. Vickovic S. Braine C. Kang K. Mollbrink A. (2019). Spatiotemporal dynamics of molecular pathology in amyotrophic lateral sclerosis. Science 364 (6435), 89–93. 10.1126/science.aav9776 30948552

[B53] Misra A. Baker C. D. Pritchett E. M. Burgos Villar K. N. Ashton J. M. Small E. M. (2021). Characterizing neonatal heart maturation, regeneration, and scar resolution using spatial transcriptomics. J. Cardiovasc. Dev. Dis. 9 (1), 1. 10.3390/jcdd9010001 35050211 PMC8779463

[B54] Mistry R. M. Singh P. K. Mancini M. G. Stossi F. Mancini M. A. (2020). Single cell analysis of transcriptionally active alleles by single molecule FISH. J. Vis. Exp. JoVE 2020 (163), 1–15. 10.3791/61680 PMC8549401 33016938

[B55] Moffitt J. R. Bambah-Mukku D. Eichhorn S. W. Vaughn E. Shekhar K. Perez J. D. (2018). Molecular, spatial and functional single-cell profiling of the hypothalamic preoptic region. Sci. (New York, N.Y.) 362 (6416), eaau5324. 10.1126/SCIENCE.AAU5324 PMC6482113 30385464

[B56] Moffitt J. R. Hao J. Bambah-Mukku D. Lu T. Dulac C. Zhuang X. (2016). High-performance multiplexed fluorescence *in situ* hybridization in culture and tissue with matrix imprinting and clearing. Proc. Natl. Acad. Sci. U. S. A. 113 (50), 14456–14461. 10.1073/pnas.1617699113 27911841 PMC5167177

[B57] Moffitt J. R. Hao J. Wang G. Chen K. H. Babcock H. P. Zhuang X. (2016). High-throughput single-cell gene-expression profiling with multiplexed error-robust fluorescence *in situ* hybridization. Proc. Natl. Acad. Sci. U. S. A. 113 (39), 11046–11051. 10.1073/pnas.1612826113 27625426 PMC5047202

[B58] Moffitt J. R. Lundberg E. Heyn H. (2022). The emerging landscape of spatial profiling technologies. Nat. Rev. Genet. 23 (12), 741–759. 10.1038/s41576-022-00515-3 35859028

[B59] Morgan J. T. Fink G. R. Bartel D. P. (2019). Excised linear introns regulate growth in yeast. Nature 565 (7741), 606–611. 10.1038/S41586-018-0828-1 30651636 PMC6464110

[B60] Moses L. Pachter L. (2022). Museum of spatial transcriptomics. Nat. Methods 19 (5), 534–546. 10.1038/s41592-022-01409-2 35273392

[B61] Osterhout J. A. Kapoor V. Eichhorn S. W. Vaughn E. Moore J. D. Liu D. (2022). A preoptic neuronal population controls fever and appetite during sickness. Nature 606 (7916), 937–944. 10.1038/S41586-022-04793-Z 35676482 PMC9327738

[B62] Park C. Owusu-Boaitey K. E. Valdes G. M. Reddien P. W. (2023). Fate specification is spatially intermingled across planarian stem cells. Nat. Commun. 14 (1), 7422. 10.1038/S41467-023-43267-2 37973979 PMC10654723

[B63] Patel A. P. Tirosh I. Trombetta J. J. Shalek A. K. Gillespie S. M. Wakimoto H. (2014). Single-cell RNA-seq highlights intratumoral heterogeneity in primary glioblastoma. Sci. (New York, N.Y.) 344 (6190), 1396–1401. 10.1126/SCIENCE.1254257 PMC4123637 24925914

[B64] Pita-Juarez Y. Karagkouni D. Kalavros N. Melms J. C. Niezen S. Delorey T. M. (2022). A single-nucleus and spatial transcriptomic atlas of the COVID-19 liver reveals topological, functional, and regenerative organ disruption in patients. BioRxiv Prepr. Serv. Biol. 10.1101/2022.10.27.514070 PMC11907808 40087773

[B65] Raj A. van den Bogaard P. Rifkin S. A. van Oudenaarden A. Tyagi S. (2008). Imaging individual mRNA molecules using multiple singly labeled probes. Nat. Methods 5 (10), 877–879. 10.1038/NMETH.1253 18806792 PMC3126653

[B66] Rao A. Barkley D. França G. S. Yanai I. (2021). Exploring tissue architecture using spatial transcriptomics. Nature 596 (7871), 211–220. 10.1038/S41586-021-03634-9 34381231 PMC8475179

[B67] Rodriques S. G. Stickels R. R. Goeva A. Martin C. A. Murray E. Vanderburg C. R. (2019). Slide-seq: a scalable technology for measuring genome-wide expression at high spatial resolution. Science 363, 1463–1467. 10.1126/SCIENCE.AAW1219 30923225 PMC6927209

[B68] Schermelleh L. Ferrand A. Huser T. Eggeling C. Sauer M. Biehlmaier O. (2019). Super-resolution microscopy demystified. Nat. Cell. Biol. 21 (1), 72–84. 10.1038/s41556-018-0251-8 30602772

[B69] Shendure J. Porreca G. J. Reppas N. B. Lin X. McCutcheon J. P. Rosenbaum A. M. (2005). Accurate multiplex polony sequencing of an evolved bacterial genome. Sci. (New York, N.Y.) 309 (5741), 1728–1732. 10.1126/SCIENCE.1117389 16081699

[B70] Shi H. Gao L. Kirby N. Shao B. Shan X. Kudo M. (2023). Clearance of VWF by hepatic macrophages is critical for the protective effect of ADAMTS13 in sickle cell anemia mice. Blood 143, 1293–1309. 10.1182/BLOOD.2023021583 PMC10997916 38142410

[B71] Sounart H. Lázár E. Masarapu Y. Wu J. Várkonyi T. Glasz T. (2023). Dual spatially resolved transcriptomics for human host–pathogen colocalization studies in FFPE tissue sections. Genome Biol. 24 (1), 237. 10.1186/S13059-023-03080-Y 37858234 PMC10588020

[B72] Ståhl P. L. Salmén F. Vickovic S. Lundmark A. Navarro J. F. Magnusson J. (2016). Visualization and analysis of gene expression in tissue sections by spatial transcriptomics. Science 353 (6294), 78–82. 10.1126/science.aaf2403 27365449

[B73] Stickels R. R. Murray E. Kumar P. Li J. Marshall J. L. Di Bella D. J. (2021). Highly sensitive spatial transcriptomics at near-cellular resolution with Slide-seqV2. Nat. Biotechnol. 39, 313–319. 10.1038/S41587-020-0739-1 33288904 PMC8606189

[B74] Stur E. Corvigno S. Xu M. Chen K. Tan Y. Lee S. (2022). Spatially resolved transcriptomics of high-grade serous ovarian carcinoma. IScience 25 (3), 103923. 10.1016/J.ISCI.2022.103923 35252817 PMC8891954

[B75] Su J. H. Zheng P. Kinrot S. S. Bintu B. Zhuang X. (2020). Genome-scale imaging of the 3D organization and transcriptional activity of chromatin. Cell. 182 (6), 1641–1659. 10.1016/J.CELL.2020.07.032 32822575 PMC7851072

[B76] Sun S. Jin C. Si J. Lei Y. Chen K. Cui Y. (2021). Single-cell analysis of ploidy and the transcriptome reveals functional and spatial divergency in murine megakaryopoiesis. Blood 138 (14), 1211–1224. 10.1182/BLOOD.2021010697 34115843 PMC8499048

[B77] Tian L. Chen F. Macosko E. Z. (2022). The expanding vistas of spatial transcriptomics. Nat. Biotechnol. 41 (6), 773–782. 10.1038/s41587-022-01448-2 36192637 PMC10091579

[B78] Tillberg P. W. Chen F. (2019). Expansion microscopy: scalable and convenient super-resolution microscopy. Annu. Rev. Cell. Dev. Biol. 35, 683–701. 10.1146/ANNUREV-CELLBIO-100818-125320 31424964

[B79] Valouev A. Ichikawa J. Tonthat T. Stuart J. Ranade S. Peckham H. (2008). A high-resolution, nucleosome position map of *C. elegans* reveals a lack of universal sequence-dictated positioning. Genome Res. 18 (7), 1051–1063. 10.1101/GR.076463.108 18477713 PMC2493394

[B80] Vandereyken K. Sifrim A. Thienpont B. Voet T. (2023). Methods and applications for single-cell and spatial multi-omics. Nat. Rev. Genet. 24 (8), 494–515. 10.1038/S41576-023-00580-2 36864178 PMC9979144

[B81] Van de Velde L. A. Kaitlynn Allen E. Crawford J. C. Wilson T. L. Guy C. S. Russier M. (2021). Neuroblastoma Formation requires unconventional CD4 T cells and arginase-1-dependent myeloid cells. Cancer Res. 81 (19), 5047–5059. 10.1158/0008-5472.CAN-21-0691 34301764 PMC8488023

[B82] Vickovic S. Schapiro D. Carlberg K. Lötstedt B. Larsson L. Hildebrandt F. (2022). Three-dimensional spatial transcriptomics uncovers cell type localizations in the human rheumatoid arthritis synovium. Commun. Biol. 5 (1), 129–786. 10.1038/S42003-022-03050-3 35149753 PMC8837632

[B83] Voelkerding K. V. Dames S. A. Durtschi J. D. (2009). Next-generation sequencing: from basic research to diagnostics. Clin. Chem. 55 (4), 641–658. 10.1373/CLINCHEM.2008.112789 19246620

[B84] Vu T. Vallmitjana A. Gu J. La K. Xu Q. Flores J. (2022). Spatial transcriptomics using combinatorial fluorescence spectral and lifetime encoding, imaging and analysis. Nat. Commun. 13 (1), 169. 10.1038/S41467-021-27798-0 35013281 PMC8748653

[B85] Wang C. Lu T. Emanuel G. Babcock H. P. Zhuang X. (2019). Imaging-based pooled CRISPR screening reveals regulators of lncRNA localization. Proc. Natl. Acad. Sci. U. S. A. 166 (22), 10842–10851. 10.1073/pnas.1903808116 PMC6561216 31085639

[B86] Wang G. Moffitt J. R. Zhuang X. (2018). Multiplexed imaging of high-density libraries of RNAs with MERFISH and expansion microscopy. Sci. Rep. 8 (1), 4847. 10.1038/s41598-018-22297-7 29555914 PMC5859009

[B87] Wang X. Allen W. E. Wright M. A. Sylwestrak E. L. Samusik N. Vesuna S. (2018). Three-dimensional intact-tissue sequencing of single-cell transcriptional states. Sci. (New York, N.Y.) 361 (6400), eaat5691. 10.1126/SCIENCE.AAT5691 PMC6339868 29930089

[B88] Wassie A. T. Zhao Y. Boyden E. S. (2019). Expansion microscopy: principles and uses in biological research. Nat. Methods 16 (1), 33–41. 10.1038/S41592-018-0219-4 30573813 PMC6373868

[B89] Williams C. G. Lee H. J. Asatsuma T. Vento-Tormo R. Haque A. (2022). An introduction to spatial transcriptomics for biomedical research. Genome Med. 14 (1), 68. 10.1186/S13073-022-01075-1 35761361 PMC9238181

[B90] Xia C. Babcock H. P. Moffitt J. R. Zhuang X. (2019). Multiplexed detection of RNA using MERFISH and branched DNA amplification. Sci. Rep. 9 (1), 7721. 10.1038/s41598-019-43943-8 31118500 PMC6531529

[B91] Xia C. Fan J. Emanuel G. Hao J. Zhuang X. (2019). Spatial transcriptome profiling by MERFISH reveals subcellular RNA compartmentalization and cell cycle-dependent gene expression. Proc. Natl. Acad. Sci. U. S. A. 116 (39), 19490–19499. 10.1073/pnas.1912459116 31501331 PMC6765259

[B92] Yao Z. van Velthoven C. T. J. Kunst M. Zhang M. McMillen D. Lee C. (2023). A high-resolution transcriptomic and spatial atlas of cell types in the whole mouse brain. Nature 624 (7991), 317–332. 10.1038/s41586-023-06812-z 38092916 PMC10719114

[B93] Zhang M. Eichhorn S. W. Zingg B. Yao Z. Cotter K. Zeng H. (2021). Spatially resolved cell atlas of the mouse primary motor cortex by MERFISH. Nature 598 (7879), 137–143. 10.1038/S41586-021-03705-X 34616063 PMC8494645

[B94] Zhang M. Pan X. Jung W. Halpern A. Eichhorn S. W. Lei Z. (2023). A molecularly defined and spatially resolved cell atlas of the whole mouse brain. BioRxiv Prepr. Serv. Biol. 10.1101/2023.03.06.531348 PMC10719103 38092912

[B95] Zhao Y. Bucur O. Irshad H. Chen F. Weins A. Stancu A. L. (2017). Nanoscale imaging of clinical specimens using pathology-optimized expansion microscopy. Nat. Biotechnol. 35 (8), 757–764. 10.1038/NBT.3892 28714966 PMC5548617

[B96] Zhu J. Fan Y. Xiong Y. Wang W. Chen J. Xia Y. (2022). Delineating the dynamic evolution from preneoplasia to invasive lung adenocarcinoma by integrating single-cell RNA sequencing and spatial transcriptomics. Exp. Mol. Med. 54 (11), 2060–2076. 10.1038/S12276-022-00896-9 36434043 PMC9722784

[B97] Zhuang X. (2021). Spatially resolved single-cell genomics and transcriptomics by imaging. Nat. Methods 18 (1), 18–22. 10.1038/S41592-020-01037-8 33408406 PMC9805800

